# Dual nematode infection in *Brassica nigra* affects shoot metabolome and aphid survival in distinct contrast to single-species infection

**DOI:** 10.1093/jxb/erae364

**Published:** 2024-08-29

**Authors:** Jessil Ann Pajar, Pius Otto, April Lyn Leonar, Stefanie Döll, Nicole M van Dam

**Affiliations:** Leibniz Institute for Vegetable and Ornamental Crops (IGZ) e.V., Großbeeren, Germany; Max Planck Institute for Chemical Ecology, Jena, Germany; Molecular Interactions Ecology, German Centre for Integrative Biodiversity Research (iDiv), Jena–Halle–Leipzig, Germany; Institute of Biodiversity, Friedrich Schiller University, Jena, Germany; Molecular Interactions Ecology, German Centre for Integrative Biodiversity Research (iDiv), Jena–Halle–Leipzig, Germany; Molecular Interactions Ecology, German Centre for Integrative Biodiversity Research (iDiv), Jena–Halle–Leipzig, Germany; Molecular Interactions Ecology, German Centre for Integrative Biodiversity Research (iDiv), Jena–Halle–Leipzig, Germany; Leibniz Institute for Vegetable and Ornamental Crops (IGZ) e.V., Großbeeren, Germany; Molecular Interactions Ecology, German Centre for Integrative Biodiversity Research (iDiv), Jena–Halle–Leipzig, Germany; Institute of Biodiversity, Friedrich Schiller University, Jena, Germany; The James Hutton Institute, UK

**Keywords:** Flavonoids, glucosinolates, hydroxycinnamic acids, metabolomics, plant–herbivore interaction, root–shoot interactions, simultaneous herbivory

## Abstract

Previous studies showed that aphid performance was compromised on *Brassica nigra* infected by root-lesion nematodes (*Pratylenchus penetrans*, Pp), but less, or positively influenced by root-knot nematode (*Meloidogyne* spp.) infection. These experiments were on single-species nematode infections, but roots can be infected naturally with several nematode species simultaneously. We performed greenhouse assays to assess the effects of single [*Meloidogyne incognita* (Mi) or Pp] and concurrent (MP) nematode infections on aphid performance. Using targeted and untargeted profiling of leaf and phloem metabolomes, we examined how single and concurrent nematode infections affect shoot metabolomes, and elucidated the possible consequences for aphid performance. We found that the metabolic response to double-infection is different from that to single-species infections. Moreover, Mi and Pp infections triggered discrete changes in *B. nigra* leaf and phloem metabolic profiles. Both Pp and MP infections reduced aphid survival, suggesting that the biological effect could primarily be dominated by Pp-induced changes. This concurred with increased indole glucosinolates and hydroxycinnamic acid levels in the leaves, in particular the putative involvement of salicylic acid-2-*O*-β-d-glucoside. This study provides evidence that concurrent infection by different nematode species, as is common in natural environments, is associated with distinct changes in aboveground plant metabolomes, which are linked to differences in the survival of an aboveground herbivore.

## Teaser

Concurrent infection of *Brassica nigra* by two different nematode species led to distinct changes in leaf and phloem metabolomes, which was linked to differences in aphid survival.

## Introduction

Plants are commonly confronted with several species of herbivores feeding on various tissues at the same time. Above- and belowground herbivores may interact via systemic induction of plant defenses triggered as a response to each herbivore (Wondafrash *et al.*, 2013; Mbaluto *et al.*, 2020; Sun *et al.*, 2022). The initial responses, usually induced by the first-arriving herbivore, alter plant chemistry in the damaged tissue and may systemically influence metabolic processes in undamaged tissues (Erb *et al.*, 2011). This systemic response may protect uninfected plant parts from further damage by reducing the performance of a later-arriving herbivore feeding on a different plant part. Hence, despite spatial separation, herbivores that share the same host may influence each other via plant-mediated signaling interactions (Soler *et al.*, 2008; Hoysted *et al.*, 2017; van Dam *et al.*, 2018; Karssemeijer *et al.*, 2020; Sun *et al.*, 2022).

Plant-parasitic nematodes (PPNs) are ubiquitous, predominantly root-feeding roundworms that cause extensive agricultural damage (Nicol *et al.*, 2011; Jones *et al.*, 2013; Tileubayeva *et al.*, 2021). PPNs accounted for annual yield losses equivalent to at least 173 billion USD worldwide (Kantor *et al.*, 2022). Two of the most economically important PPN groups that are distributed globally are root-knot nematodes (RKN, *Meloidogyne* spp.) and root-lesion nematodes (RLN, *Pratylenchus* spp.) (Nicol *et al.*, 2011; Jones *et al.*, 2013; Kantor *et al.*, 2022). RKNs are sedentary endoparasites that stealthily feed in the roots. Their infection mechanism involves penetrating the root epidermis and establishing their feeding site in the vascular cylinder. The infective second-stage juveniles (J2) move in between cells to avoid damaging other cells along the way (Kyndt *et al.,* 2012a; Bartlem *et al.*, 2014). The establishment of the feeding site and the biotrophic feeding mechanism of RKNs cause extensive changes in plant cellular and metabolic processes, mostly attributed to a huge array of effector proteins injected by the feeding nematodes into the plant (Kyndt *et al.*, 2012b; Rutter *et al.*, 2022; Mitchum *et al.*, 2023). RLNs, on the other hand, are migratory endoparasites that damage the roots by continuously moving through cells in between feeding, thereby causing severe root necrosis, which renders infected tissues more vulnerable to secondary infections (Sijmons *et al.*, 1994; Fosu-Nyarko and Jones, 2016).

While PPNs mostly damage the roots, they are also known to systemically induce defenses in aboveground plant parts (Bezemer and van Dam, 2005; Hofmann *et al.*, 2010; van Dam *et al.*, 2018; Shi *et al.*, 2022). Kyndt *et al.* (2012a) reported the systemic reprogramming of defense-related genes in rice shoots when the plants were previously exposed to either sedentary or migratory endoparasitic nematodes. Gene expression analysis revealed different mechanisms by which these two nematodes of different feeding strategies systemically induce plant defenses. The migratory nematode *Hirshmaniella oryzae* induced genes associated with the phenylpropanoid pathway and ethylene and methyl jasmonate biosynthesis, as well as rice pathogenesis-related genes at 3 d post-inoculation. These same genes were down-regulated in plants infected with the RKN *Meloidogyne graminicola*. This systemic reprogramming of defense-related genes and metabolites in aboveground plant tissues during nematode feeding can affect the plant’s response to aboveground herbivores and pathogens (van Dam *et al.*, 2018; Mbaluto *et al.*, 2021; Shi *et al.*, 2022). For instance, *M. graminicola* was reported to predispose infected rice to further damage by the fungal pathogen *Magnaporthe oryzae*. This was attributed to altered auxin activity and weakened general primary and secondary metabolism after *M. graminicola* pre-infection, resulting in significantly faster rice blast disease progression (Kyndt *et al.*, 2017). In another study, the slow growth and reduced pupation rate of the cabbage white butterfly (*Pieris rapae*) were attributed to changes in leaf glucosinolate and phenolic concentrations as a consequence of *P. penetrans* infections in the roots (van Dam *et al.*, 2005).

The black mustard plant (*Brassica nigra*) is simultaneously attacked by PPNs of several genera belowground and by the aphid *Brevicoryne brassicae* aboveground (Hol *et al.*, 2016). Previous studies showed that aphid population growth is less- or positively affected on plants infected with *Meloidogyne* spp., whereas it was lower on plants infected with *Pratylenchus penetrans* (Hol *et al.*, 2013, 2016; van Dam *et al.*, 2018). It was suggested that phytohormonal crosstalk between jasmonic acid (JA) and salicylic acid (SA) facilitated aphid-induced responses in plants with prior nematode infection. The presence of *Meloidogyne hapla* in the roots led to systemic up-regulation of the JA biosynthesis genes *VSP2* and *MYC2* in the leaves, 9 d after aphid infection (van Dam *et al.*, 2018). As aphids are mostly susceptible to increased SA-induced defenses, plant responses to nematode infections belowground influenced the response to aphid infection aboveground (Züst and Agrawal, 2016; van Dam *et al.*, 2018).

The observed effects of belowground nematode infections on aboveground-feeding aphids imply that the induction of plant defense against nematodes is not just localized in the roots, but triggers systemic signaling in aboveground tissues. Many defense compounds such as glucosinolates (Chen *et al.*, 2001), alkaloids (Hartmann *et al.*, 1989; Erb *et al.*, 2009), and flavonoids (Buer *et al.*, 1988), as well as signaling molecules like SA derivatives (Ratzinger *et al.*, 2009) and sugars (Koenig and Hoffmann-Benning, 2020), can be transported over long distances within the plant. The phloem provides a highway for signaling molecules and defense compounds, thereby serving important functions for both resource (re)distribution and plant defense (Van Bel and Gaupels, 2004; Dinant and Lemoine, 2010; Koenig and Hoffmann-Benning, 2020; Broussard *et al.*, 2023). Changes in phloem chemistry may directly affect aphids as they are phloem feeders. Therefore, PPN-induced changes in amino acid and sugar concentrations, as well as increased defense compounds in the phloem, may severely impact aphid performance (Sandström and Pettersson, 1994; Pescod *et al.*, 2007; Dinant *et al.*, 2010).

While it has been documented that single-species nematode infections affect plant–aphid interactions due to differences in nematode feeding strategies, nematodes are known to co-infect roots in nature (Estores and Chen, 1972; Melakeberhan and Dey, 2003; Fontana *et al.*, 2015; Mokrini *et al.*, 2018). In a survey evaluating the occurrence of PPNs infecting *B. nigra*, Hol *et al.* (2016) found that both *P. penetrans* and *Meloidogyne* spp. dominate the nematode populations on roots of field-grown *B. nigra*. Studies on concurrent aboveground herbivory, for example by caterpillars and aphids, showed that two herbivores with different feeding strategies can affect each other’s performance via systemic-induced responses when feeding on the same host (Ali and Agrawal, 2014; Kroes *et al.*, 2017). Additionally, aboveground herbivores and the impact of their interaction also affect the interaction between plants and belowground organisms (Bezemer and van Dam, 2005; Rasmann, 2022). The interaction between co-inhabiting nematode species belowground may likewise affect local and systemic responses, thereby affecting aboveground herbivores feeding on the same host. Such interactive effects of multiple nematode species on aboveground herbivores via systemically induced responses are scarcely studied.

In this study, we aimed to identify plant-mediated mechanisms underlying differences in aphid performance in the presence of single and concurrent nematode infections. We used two root-feeding PPN species of different feeding types, the RKN *Meloidogyne incognita* (Mi) and the RLN *Pratylenchus penetrans* (Pp), as well as the aphid *Brevicoryne brassicae*. We hypothesized that each nematode species will induce discrete changes in shoot metabolic profiles, whereas simultaneous nematode infection will elicit an attenuated defense response, for example due to negative cross-talk between the SA and JA pathways induced by each species. Furthermore, we postulated that this will lead to metabolic profile shifts in leaves and phloem, which will differentially influence aphid performance and aphid-induced plant responses aboveground. We looked into putative plant defense and signaling compounds that may be enhanced or suppressed in plants infested with both nematodes and aphids, encompassing early and late infection time points.

## Materials and methods

### Biological materials

#### Plant conditions

*Brassica nigra* L. seeds bulk-collected from a wild population at Elderveld, Arnhem, the Netherlands in 2005 were germinated in water-soaked glass beads in plastic containers. The containers were covered with transparent plastic lids and kept in a climate chamber in a 16:8 h (light: dark) photoperiod at 20:16 °C (day: night). The seeds were allowed to germinate for ~10 d. Plant pots were prepared and maintained following the protocol described by van Dam *et al.* (2003). Briefly, before transplanting each pot was filled with 2.5 litres of dry, previously heat-treated sand (90 °C for 1 h) and supplied with 200 ml tap water. The plants were grown under the following greenhouse conditions: 16:8 h photoperiod with minimum light intensity of 300 µmol m^−2^ s^−1^; mean temperature of 25 °C; and 60–80% relative humidity. The plants were supplied with 100 ml of half-strength 3P Hoagland solution per week to avoid P deficiency symptoms from which *B. nigra* may suffer (van Dam *et al.*, 2003). Substrate moisture was maintained at 12% using an ML3 Theta kit (Delta-T Devices Ltd, Cambridge, UK). The developmental stage of *B. nigra* was monitored following the universal BBCH scale (Lancashire *et al.*, 1991).

#### Aphid culture

The cabbage aphid (*Brevicoryne brassicae*) was obtained from the Department of Biochemistry, Max Planck Institute for Chemical Ecology, Jena, Germany. The starting colony was first maintained on *Brassica oleracea* and later transferred to *B. nigra*. The aphid-infected plants were placed in net cages inside a growth chamber under the following conditions: 16:8 h (light: dark) photoperiod; 22:18 °C temperature (day: night); 40:70% relative humidity; and minimum light intensity of 220:0 µmol m^−2^ s^−1^. Ontogenetically synchronized aphids were used in every experiment.

#### Nematode cultures

*Pratylenchus penetrans* was provided by the Plant Science Research Unit, Research Institute for Agriculture, Fisheries, and Food (ILVO), Merelbeke, Belgium. The culture was maintained on carrot discs at 25 ± 1 °C. For the experimental set-up with plants used for phloem metabolomics, we used *P. penetrans* acquired from the Field Crops Research Unit, Wageningen University and Research, The Netherlands. *Meloidogyne incognita* was provided by Bejo, Warmenhuizen, the Netherlands and was maintained on *Solanum lycopersicum* cv. ‘Moneymaker’. All stages of *P. penetrans* and J2s of *M. incognita* were extracted from their respective cultures using a modified Baermann technique (Hooper *et al.*, 2005). Aliquots containing 100 nematodes per millilitre plus Tween-20 surfactant (0.04% v/v) were prepared and used for inoculations.

### Nematode treatment

To investigate the effect of belowground nematode infection on aphid performance, infection assays were set up with the following treatment groups: Control, *M. incognita* only (Mi), *P. penetrans* only (Pp), and *M. incognita + P. penetrans* inoculated simultaneously (MP). Four-week-old (BBCH 32) *B. nigra* plants were inoculated with 2 ml of nematode suspension, containing 200 nematodes in the infective stage in water–Tween-20 solution each. The same number of nematodes was applied in MP treatments with a combination of 100 J2s of *M. incognita* and 100 infective stages of *P. penetrans* in 2 ml solution. Control plants were mock-inoculated with the same amount of water–Tween-20 solution. The nematode suspension was introduced into a small hole near the roots and were allowed to infect for 7 d before subjecting the plants to assays.

After aphid counting or sampling of the plant materials needed, the roots of infected plants were thoroughly washed and examined for the presence of galls or root lesions to confirm the success of nematode treatments (Supplementary Fig. S1).

### Aphid performance assessment

We used a two-time-point approach to assess the effects of belowground nematode infection on the critical phases of plant–aphid interactions. This encompasses the response of *B. nigra* to the aphids’ initial establishment and early development (survival, early time point), as well as sustained feeding and reproduction (population growth, late time point). This approach also allowed us to look into the systemic response of *B. nigra* to the early and later feeding stages of the nematodes, which are characterized by different systemic induced responses (Mbaluto *et al.*, 2020). The early time point [10 days post-nematode inoculation (dpni); 3 days post-aphid inoculation (dpai)] considers the effects of the different feeding strategies of the PPNs and their effect on initial aphid establishment and survival. The late time point (26 dpni, 19 dpai) covers a period of two to three generations of aphids, which allowed us to assess the effect of the PPNs on aphid population growth.

#### Aphid survival

**Fig. 1.**
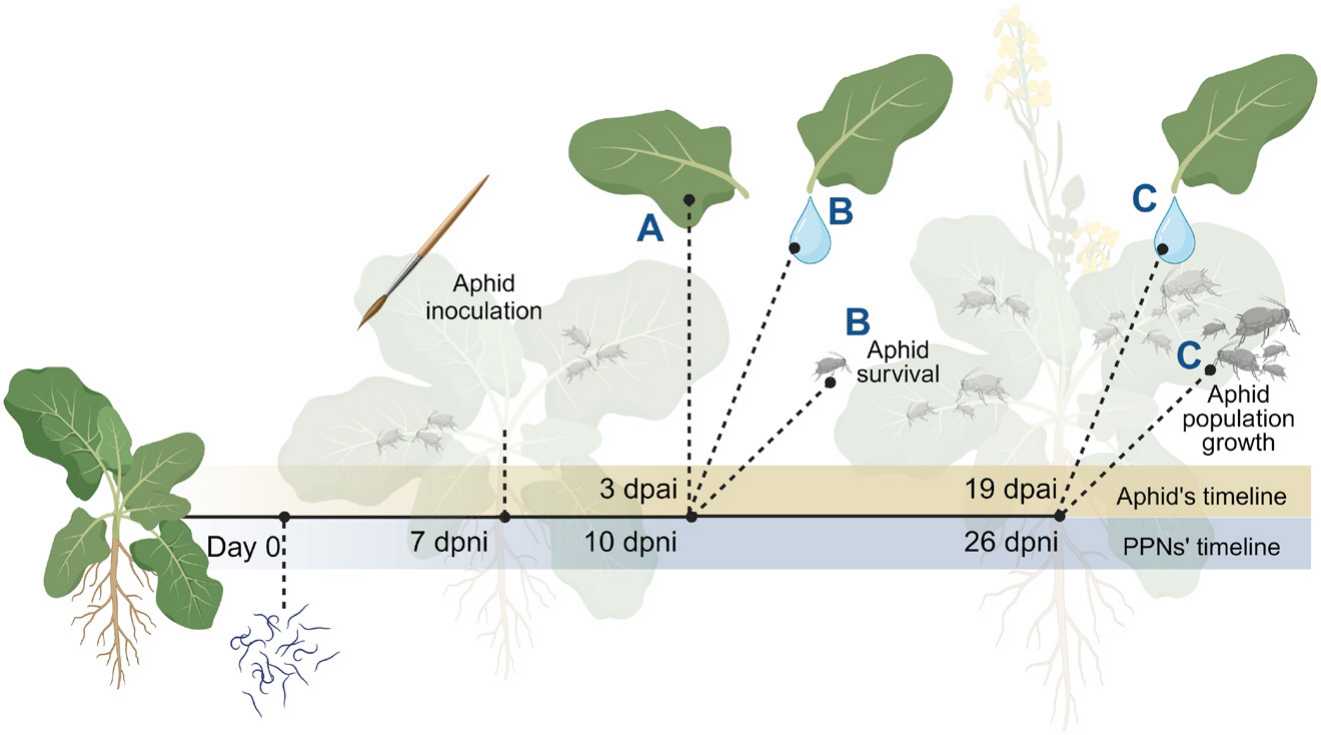
Diagram showing the experimental timeline and the nematode and aphid infection time points. Each experimental set-up (set-ups A–C) was performed on 4-week-old *Brassica nigra* plants. Single-species (*Meloidogyne incognita*, *Pratylenchus penetrans*) and concurrent nematode inoculations marked the start of the assays (day 0). On the 7th day post-nematode infection (7 dpni), 2-day-old aphid nymphs were inoculated on the leaves. On the third day post-aphid inoculation (3 dpai), leaf samples were taken from the plants in experimental set-up A, while phloem exudates and aphid survival data were acquired from the plants in experimental set-up B. In a similar set-up, set-up C, the aphids were allowed to feed and reproduce for 16 d further. At 19 dpai (corresponding to 26 dpni), the aphids were counted and removed, and then the second set of phloem samples were collected. Created with BioRender.com.

Aphid survival was assessed to determine whether nematode treatments affect early aphid development. At 1 week post-nematode inoculation, 15 2-day-old nymphs were inoculated onto five fully developed leaves per plant of all designated treatments (control, Mi, MP, Pp). The aphids were introduced to all plants by gently transferring them onto the leaf surface using a wet fine brush. The aphids were restricted to feeding only on the assigned plant by enclosing the shoot in a 20 × 27 cm organza bag, tied around the upper part of the pot. After 3 d of feeding, the number of aphids alive within the enclosed potted plants was recorded (Fig. 1B).

#### Aphid population growth

To evaluate how each nematode treatment affected aphid performance over a longer period of feeding and reproduction, we performed aphid population growth analysis in a no-choice experiment. Ten *B. nigra* plants (BBCH 32) were assigned to the previously defined treatments. On the 7th d after nematode inoculation, 15 2-day-old *B. brassicae* nymphs were placed on fully unfolded leaves per plant. A 50 × 65 cm organza bag was used to restrict the aphids in the assigned plant. Aphid population growth, as reflected by the total number of nymphs and adults per plant, was recorded at 19 dpai (Fig. 1C). Briefly, the aphids infecting the leaves selected for phloem exudation were gently removed by brushing them into a labelled paper bag. The rest of the plant shoot along with the aphids on other leaves and the stems were collected in the same paper bag. The collected aphids were then oven dried at 40 °C for 3 d. The dried aphids were counted under a stereomicroscope, defining the total number of aphids per plant.

### Leaf sampling for untargeted metabolomics

In a separate but similar set-up, 10 2-day-old nymphs were placed on the fourth and fifth leaves of the plants assigned to aphid treatment. These aphid-inoculated leaves were enclosed in organza bags to keep aphids from moving to other plant parts. The fourth and fifth leaves of plants that were not inoculated with aphids were similarly enclosed in organza bags to control for the effect of bagging in the analysis. Two leaf samples (fourth and fifth leaves) per plant were collected for targeted and non-targeted metabolomic analyses. The bags and aphids were gently removed and the leaves were sampled at 3 dpai, which coincided with the previously described aphid survival analysis timeline. We collected 6–10 leaf samples per treatment group. Leaf samples were flash-frozen in liquid nitrogen, stored at −80 °C, freeze-dried, and finely pulverized, then stored at room temperature until metabolite extraction.

### EDTA-facilitated phloem exudate collection

Collection of phloem exudates followed acquisition of the data for aphid survival and population growth (Fig. 1B, C). Phloem samples were collected following the protocol described by Xu *et al.* (2019). This procedure was optimized to contain predominantly phloem exudates and was found to be efficient in assessing the phloem components of several plant samples (Tetyuk *et al*., 2013; Xu *et al.*, 2019; Ogden *et al.*, 2020; Blicharz *et al.*, 2021). Briefly, the leaves were cut at the base of their petiole and submerged into 8 mM EDTA solution on a Petri dish. The EDTA-submerged leaves were stored in a dark, humid chamber at 20 °C. One hour later, the EDTA was washed off thoroughly from the leaves with ultrapure water and leaves were then submerged in ultrapure water, where they were allowed to exude in the previously mentioned chamber. After 12 h of exudation, the leaves were removed, oven-dried and weighed. The phloem exudates were flash-frozen in liquid nitrogen and freeze-dried. This collected sap is henceforth referred to as the phloem exudate.

### Untargeted leaf metabolomics via LC-MS coupled with MS/MS

Liquid chromatography–time of flight mass spectrometry (LC-ToF-MS) was used to characterize the metabolic profiles of the samples. Extraction of leaf metabolites followed as in Weinhold *et al.* (2022). LC-quadrupole (q) ToF-MS analysis was conducted on an UltiMate 3000 standard ultra-high-performance liquid chromatography system (Thermo Scientific) with an Acclaim 120 rapid separation liquid chromatography column (150 mm×2.1 mm, particle size 2.2 μm, Thermo Fisher Scientific, Waltham, MA, USA) using the following: gradient and flow rate of 0.4 ml min^–1^: 0–1 min, isocratic 95% A [water/formic acid 99.9/0.1 (v/v %)], 5% B [acetonitrile/formic acid 99.9/0.1 (v/v %)]; 1–2 min, linear from 5% to 20% B; 3–8 min, linear from 20% to 25% B; 8–16 min, linear from 25% to 95% B; 16–18 min, isocratic 95% B; 18–18.01 min, linear from 95% to 5% B; 18.01–20 min, isocratic 5% B. Data were recorded from 0 min to 18 min. The injection volume was 10 μl. Eluted compounds were detected from *m*/*z* 90 to 1600 at a spectrum rate of 5 Hz (line spectra only) using electrospray ionization ultra-high-resolution qToF-MS (maXis impact, Bruker Daltonics) in positive ion mode with data-dependent collision-induced dissociation (Auto-MSMS mode). The instrument settings are described in detail by Weinhold *et al.* (2022). One quality control containing 5 μl of each sample was prepared. MM8, a mix of eight commercial standards, was also included. The blanks used were acetonitrile and 75% methanol (80% methanol for the phloem samples).

Data processing was performed with Bruker Compass MetaboScape Mass Spectrometry Software (5.0.0). Peak picking and alignment, mass recalibration, feature extraction, and grouping of isotopes, adduct, and charge states were done with the T-ReX algorithm in MetaboScape. After processing and blank feature subtraction, the dataset contained 7034 features (Supplementary Table S1, sheet 1). The resulting features with MS/MS data were annotated using an in-house analyte list. The features were then classified using SIRIUS/CANOPUS (Dührkop *et al.*, 2019, 2021; Kim *et al.*, 2021) and formatted with MetIgel v.1.0.

### Untargeted phloem metabolomic analysis

Freeze-dried phloem samples were redissolved in 1 ml 80% methanol and placed in an ultrasonic bath for 15 min at 35 °C. Afterwards, they were centrifuged at 10 000 *g* for 15 min at room temperature. The supernatant was transferred to a new 2 ml Eppendorf tube while the pellet was re-extracted with 500 μl of 80% methanol and centrifuged at 10 000 *g* for 15 min at room temperature. The two extracts were combined and concentrated using a vacuum concentrator. Each sample was normalized to the leaf dry weight. The final volume was then transferred into LC-MS vials. LC-MS analysis in positive ionization mode was performed as described above. Data processing was performed similarly to the leaf metabolome data. After blank feature subtraction, the phloem dataset contained 5931 features, of which 2900 could be classified (Supplementary Table S1, sheet 2).

### Targeted metabolite analyses

#### Glucosinolate analysis

Glucosinolates (GSLs) were extracted and analysed following the protocol of Grosser and van Dam (2017). Aliquots of 50 mg freeze-dried, ground leaf samples were weighed in 2 ml round-bottom Eppendorf tubes. GSLs were separated by HPLC (UltiMate 3000, Thermo Scientific) equipped with a photodiode array detector (Thermo Scientific Ultimate 3000 series) at 229 nm and 272 nm wavelengths. A reversed-phase Acclaim 300 C18 column (4.6 × 150 mm, 3 μm, 300 Å, Thermo Fisher Scientific) was used for separation with 100% H_2_O and 99% acetonitrile in water as solvents. Separation conditions were described in detail by Touw *et al.* (2020). Data processing was performed using Chromeleon 7.2 SR5 MUa (9624; Thermo Fisher Scientific).

#### Phytohormone quantification

To build on the existing information about the phytohormone-mediated interaction between individual PPN species and aphids, we measured the concentrations of phytohormones in the leaves. We focused particularly on SA and JA, which have been previously studied in the system. Additionally, we examined the levels of JA-Ile, a bioactive form of JA, and abscisic acid (ABA), which is known to respond to herbivory as well, thereby modulating the SA- and JA-induced responses via cross-talk (Ratzinger *et al.*, 2009). Phytohormones were extracted from 20 mg of ground lyophilized leaf samples and were quantified using a protocol modified from Machado *et al.* (2013). Twenty milligrams of pulverized dry leaf sample was mixed with 1 ml ethyl acetate containing 40 ng of deuterated internal phytohormone standards: D6-JA, D6-Ja-Ile, D6-SA, and D6-ABA. The phytohormones were measured by liquid chromatography (Acquity UPLC, Waters) coupled to a mass spectrometer (Bruker Elite EvoQ Triple quadrupole, Bremen, Germany) (LC/MS EVOQ). Separation parameters were described in detail by Mbaluto *et al.* (2020). Data processing was performed in MS data review software (Bruker MS Workstation, version 8.2). Phytohormone levels were calculated based on the peak area of the corresponding internal standard and the weight of plant material.

#### Phloem sugar analysis

The same phloem samples used in untargeted phloem metabolomics were used in the targeted analysis of quantifiable sugars in the phloem. This was performed using the method described by Sławińska *et al.* (2021), with a few modifications. Briefly, the sugars were separated on an HPLC equipped with a Corona Veo RS-charged aerosol detector (Germering, Germany). Separation was carried out using a Shodex Asahipak NH2P-50 4E 5 μm (4.6 × 250 mm) column with precolumn Asahipak NHZP-506 4A. The mobile phase of the optimized method was composed of (A) 25% water + 0.05% formic acid and (B) 75% acetonitrile + 0.05% formic acid. The flow rate was 1 ml min^−1^. The column temperature was maintained at 30 °C. The injection volume was 10 μl for both standards and phloem extracts. The chromatographic separation time was set to 22 min. Data processing was performed with Chromeleon 7.2 SR5 MUa (9624; Thermo Fisher Scientific).

### Statistical analyses

After data processing and compound annotation of the untargeted metabolomic data, the resulting feature tables, hereafter called leaf and phloem datasets, were exported to Metaboanalyst 5.0 (Pang *et al.*, 2021). Each dataset was filtered based on the interquantile range. Prior to data analysis, each dataset was log-transformed and Pareto-scaled. These feature tables were used in partial least squares discriminant analysis (PLS-DA). The lists of differentially abundant (DA) features based on pairwise comparisons between control and each of the nematode-treated plants (Mi, Pp, MP) were obtained using the Volcano Plot function in Metaboanalyst 5.0. We used volcano plots to show how many features are differentially abundant in the nematode-infected versus control plants. The DA features were determined at threshold fold change (FC) >2.0 over controls at *P*<0.05. The lists were exported to InteractiVenn (Heberle *et al.*, 2015) to create the Venn diagrams. For the features classified with NPClassifier (Kim *et al.*, 2021) in the untargeted phloem metabolomics data, PERMANOVA was performed using the ‘adonis2’ function in the R package vegan, using Bray–Curtis as distance measure. Spearman’s rank correlation test was performed using the ‘cor.test’ function of the R package *stats*.

Statistical analyses for targeted metabolomic measurements were performed using R Statistical Software (v4.1.2, 2021; R Core Team). Missing values were subject to missing value imputation using a Classification and Regression Trees (cart) algorithm embedded in the Multivariate Imputation by Chained Equations (*mice*) package in R (Van Buuren and Groothuis-Oudshoorn, 2011). Multivariate analysis of variance (MANOVA) was performed using the Manova function in the R package *car* (Fox and Weisberg, 2019) followed by univariate ANOVA type III (ezANOVA) to analyse the effect of significant predictors to each phytohormone or glucosinolate. Post-hoc analysis was performed using the Pairwise.*t*.test function in R with Bonferroni correction.

Statistical analyses for phloem sugars and aphid performance were performed using the *lme4* package in R (Bates *et al.*, 2015). Distributions and variances were assessed with QQ-plots, Shapiro–Wilk and Levene’s test. The data were log-transformed when needed to meet parametric test assumptions. The data for phloem sugar concentrations were subject to a generalized linear model (GLM), gamma distribution (link=‘log’). The aphid survival dataset is a combination of results from two independent experiments. The combined dataset was analysed using a linear mixed model with Gaussian distribution with nematode treatments (control, Mi, MP, and Pp) as the fixed factor and the different experimental set-up as the random factor. Aphid population growth data were analysed with a linear model (LM) using the log-transformed values of aphid count. Model selection was done by comparing Akaike information criterion values. Pairwise comparisons were performed using the R package *multcomp* (Hothorn *et al.*, 2008).

Boxplots represent the interquartile range with the upper line representing the 75th percentile and the lower line representing the 25th percentile. The middle line represents the median. The whiskers extend to the (upper) maximum and (lower) minimum values, illustrating the overall spread of the data within one treatment. Black dots beyond the whiskers represent the outliers. Points represent the number of biological replicates per treatment. Figures and plots were created via *ggplot2* in R and were optimized in Inkscape 1.1.1 (3bf5ae0d25, 2021-09-20, inkscape.org).

## Results

### Nematodes induced metabolic profile shifts in *B. nigra* leaves and phloem

#### Leaves

**Fig. 2.**
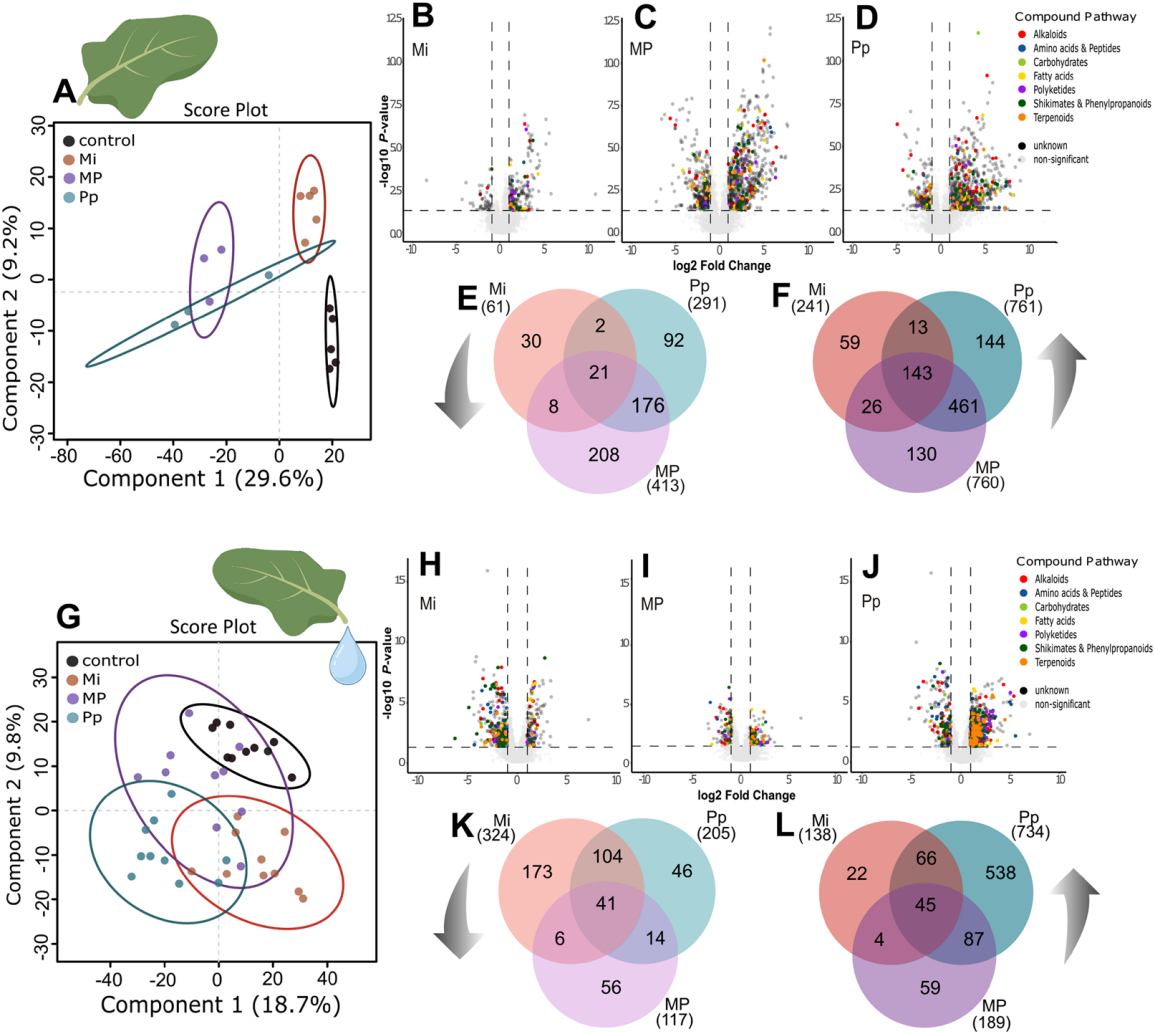
Metabolic profile of *Brassica nigra* leaves (A–F) and phloem (G–L), 10 d after single [*Meloidogyne incognita* (Mi), *Pratylenchus penetrans* (Pp)] or dual [*M. incognita* and *P. penetrans* together (MP)] nematode treatments. Metabolic profiles were constructed based on LC-qToF-MS/MS analyses in positive ionization mode. Separation of leaf (A) and phloem (G) metabolic profiles of nematode-treated plants and mock-inoculated control plants shown in 2D scores plot following partial least squares discriminant analysis (PLS-DA). Points represent biological replicates per treatment group (leaf for Mi and control *n*=5, for MP and Pp *n*=3, phloem *n*=10). Percentages along the axes indicate the variation explained by each component, and ellipses indicate 95% confidence interval. PLS-DA cross-validation was based on three components: (A) leaf metabolome: 5-fold cross-validation: accuracy=0.63, *R*^2^=0.93, *Q*^2^=0.41); (G) phloem metabolome (5-fold cross-validation: accuracy=0.56, *R*^2^=0.93, *Q*^2^=0.51). Volcano plots show differentially abundant (DA) metabolites in the leaves (B–D) and phloem (H–J) of nematode-infested versus control plants. Metabolites were considered DA based on the following thresholds: fold change >2.0, *P*≤0.05 (*t*-test). (B–D) show DA metabolites in the leaves of Mi- (B), MP- (C), and Pp- (D) treated plants. (H–J) show DA metabolites in the phloem of Mi- (H), MP- (I), and Pp- (J) treated plants. The colored points represent metabolite groups classified via NPClassifier. The number of unique and shared DA metabolites are summarized in Venn diagrams (E, F, K, L); (E, F) show the number of down- (E) and up- (F) regulated metabolites in the leaves; (K, L) show the number of down- (K) and up- (L) regulated metabolites in the phloem. Up and down arrows indicate that data are from up- and down-regulated DA features. The complete list of DA metabolites is given in Supplementary Table S1.

Partial least squares-discriminant analysis (PLS-DA) showed that leaf metabolic profiles of nematode-infected plants are dissimilar from that of control plants (5-fold cross-validation: accuracy=0.63, *R*^2^=0.93, *Q*^2^=0.41), with two components explaining 38.8% of the variation (Fig. 2A). Supplementary Fig. S2 shows the top 10 (A) and top 50 (B) features as revealed by the model’s variable importance in the projection (VIP) from the first component. This shows that there is a clear difference in metabolomic leaf profiles of Pp- and MP-infected versus those of control and Mi-infected plants. Most of the top 50 features were accumulated more in leaves of Pp- and MP-infected plants, except for the unknown compound eluting at 15.17 min with *m*/*z* 16.45071 (Supplementary Fig. S2B). Volcano plots show that the leaves of Mi-treated (Fig. 2B) plants had fewer DA features than MP- (Fig. 2C) and Pp-treated (Fig. 2D) plants. Venn diagrams showed that, out of the 1052 DA features in the leaves of Pp-infected plants, 236 were unique for Pp infection [Fig. 2E, F, Pp, 92 down (E) and 144 up (F)]. In leaves of Pp-treated plants only, tryptophan [log_2_FC=1.06, *P*<0.05, retention time (RT) 3.62 min, *m*/*z* 205.09718], *cis*-12-oxophytodienoic acid (OPDA, log_2_FC=2.290, *P*<0.005, RT 14.65 min, *m*/*z* 293.2099) and linoleic acid (log_2_FC=2.245, *P*<0.05, RT 17.48 min, *m*/*z* 281.249) were significantly accumulated, and raphanusamic acid (log_2_FC=−1.18, *P*<0.01, RT 2.94 min, *m*/*z* 163.9836) was significantly reduced (Supplementary Fig. S3).

In the Mi treatment, on the other hand, only 89 out of the 302 DA features were uniquely regulated (Fig. 2E, F, Mi, 30 down and 59 up). In addition, there were relatively few DA features that were shared by both Mi and Pp infections, comprising only 2 down- and 13 up-regulated features, which is probably due to the differences in their specific interactions with their host plants. Plants in the MP treatment group had 1173 DA features in their leaves. The 130 uniquely up-regulated DA features in MP-treated plants included the amino acid l-glutamine (log_2_FC=1.79, *P*<0.05, RT 1.05 min, *m*/*z* 147.07313). Moreover, the MP treatment uniquely reduced 208 features, among which the amino acid l-tyrosine (log_2_FC=−1.48, *P*<0.01, RT 1.48 min, *m*/*z* 182.08129) and a coumarin, scoparone (log_2_FC=−1.49, *P*<0.05, RT 8.14 min, *m*/*z* 207.06508) (Supplementary Fig. S3). The leaves of MP-infected plants shared more DA features with Pp (176 down and 461 up) than with Mi (8 down and 26 up; Fig. 2E-F). Among the annotated features, Pp and MP treatments commonly enhanced the accumulation of sinapic acid (RT 5.81 min, *m*/*z* 207.0651), yet reduced the accumulation of l-ascorbic acid (RT 1.04 min, *m*/*z* 177.03) in the leaves (Supplementary Fig. S3). Furthermore, the Venn diagrams show that there were 21 features down-regulated and 143 up-regulated by any nematode treatment (Fig. 2E, F, center), suggesting that these constitute the core-metabolic response to nematode infection. A complete list of DA features is given in Supplementary Table S1 (sheets 3–5).

#### Phloem

The influence of belowground nematode infection was also evident in the phloem metabolic profile at 10 dpni. PLS-DA showed that phloem metabolic profiles of Mi- and Pp-infected plants were dissimilar to that of control plants (Fig. 2G, 5-fold cross-validation: accuracy=0.56, *R*^2^=0.93, *Q*^2^=0.51). Similar to the leaf metabolic profiles, VIP analyses showed that phloem of Pp- and MP-infected plants had a different metabolomic profile from control and Mi-infected plants, though the similarity between Pp and MP phloem profiles was less clear than in leaves (Supplementary Fig. S4). The top 50 important features were accumulated more after Pp and MP infections except for three unknown features, in particular one eluting at 11.1 min with *m*/*z* 831.28386 (Supplementary Fig. S4B). Volcano plots showed that the phloem of Pp-infected plants had more DA features (939) than that of Mi- (462) or MP- (306) treated plants (Fig. 2H-J). The up-regulated DA features (538) uniquely associated with Pp infection included the phytohormone ABA (log_2_FC=1.04, *P*<0.01, RT 9.89 min, *m*/*z* 247.132; Supplementary Fig. S5), the amino acid phenylalanine (log_2_FC=1.0015, *P*<0.01, RT 2.54 min, *m*/*z* 166.08638) and a phenolic compound, syringic acid (log_2_FC=1.1361, *P*<0.01, RT 4.36 min, *m*/*z* 199.0603) (Supplementary Fig. S5). Mi-treated plants uniquely reduced the accumulation of several features classified as flavonoids, such as luteolin (log_2_FC=−1.2959, *P*<0.05, RT 10.03 min, *m*/*z* 287.05) (Supplementary Fig. S5). In total, Mi treatment uniquely down-regulated 173 features (Fig. 2K) and uniquely up-regulated 22 features (Fig. 2L) in the phloem. Compared with leaves, more features were shared among Mi and Pp treatments in phloem profiles (down 104, up 66). The MP treatment down-regulated 56 features whereas it up-regulated 59 in the phloem (Fig. 2K, L). The MP treatment reduced the abundance of *trans*-cinnamic acid (log_2_FC=−1.72, *P*<0.001, RT 10.56 min, *m*/*z* 131.05) in the phloem (Supplementary Fig. S5). Despite huge differences between Pp- and MP-treated plants in the numbers of DA phloem features, these two groups shared more DA phloem features (14 down and 87 up) than MP-treated plants did with Mi-treated plants (6 down and 4 up; Fig. 2K, L). Both Pp- and MP-treated plants accumulated l-histidine (RT 0.85 min, *m*/*z* 156.076) in the phloem (Supplementary Fig. S5). Also, in the phloem, there was a core metabolic response to any nematode infection consisting of 41 down-regulated and 45 up-regulated features. A complete list of DA features in the phloem is given in Supplementary Table S1 (sheets 6–8).

These results show that nematode infections belowground induced significant metabolic profile shifts in both leaves and phloem of *B. nigra.* Plants infected with both *M. incognita* and *P. penetrans* induced DA features distinct from those infected by either nematode alone.

### Consequences of nematode infections for aphid performance aboveground

**Fig. 3.**
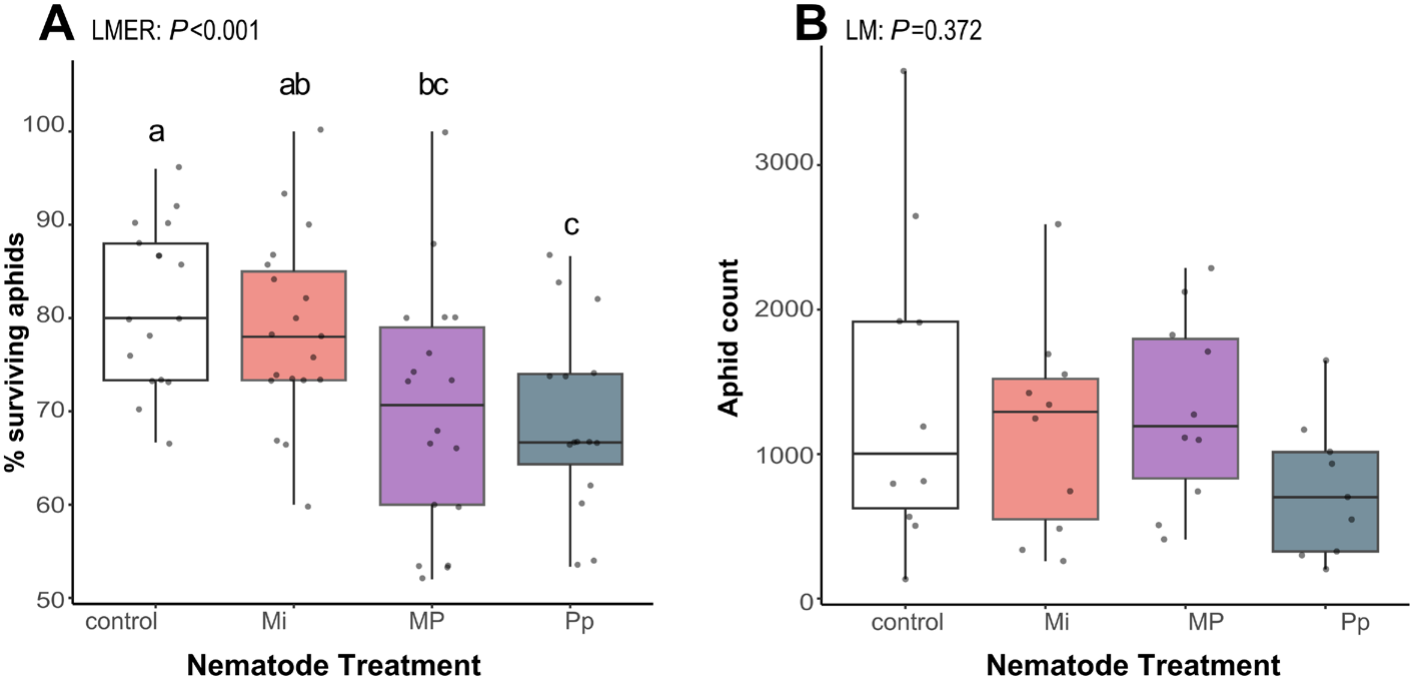
Aphid (*Brevicoryne brassicae*) performance parameters. (A) Aphid survival showing the effect of nematode treatment on mean percentage of surviving aphids after 3 d of initial feeding on plants infested with *Meloidogyne incognita* (Mi), *Pratylenchus penetrans* (Pp), and *M. incognita* and *P. penetrans* together (MP), or mock-inoculated with water–Tween-20 solution (control). (B) Aphid population growth showing the total number of aphids at 19 d after the inoculation of 15 2-day-old nymphs per plant. The number of aphids (aphid count) accounts for both survival and reproduction over an extended period of feeding. Box plots labeled with different letters are significantly different as per Tukey’s test for pairwise comparisons at *P*<0.05. Points represent the number of biological replicated per treatment (A: control, *n*=10; Mi and MP, *n*=18; Pp, *n*=15; B: control, Mi, and MP, *n*=10; Pp, *n*=9).

Aphid survival was significantly influenced by prior nematode infection (Fig. 3A; LMER: χ^2^=17.90, df=3, *P*<0.001). The mean percentages of surviving aphids were significantly lower on plants pre-infected with Pp [Tukey’s HSD post-hoc test, adjusted *P*-value (*P*_adj_)*=*0.005] or MP (*P*_adj_*=*0.007), which had 12% and 10% lower survival rates than on control plants, respectively. The percentage of surviving aphids in MP*-*treated plants was marginally lower than on Mi-treated plants (*P*_adj_*=*0.066), but did not significantly differ from those on plants Pp infection. Aphid survival in Mi-treated plants was significantly higher than on Pp-treated plants (*P*_adj_*=*0.043), but did not significantly differ from those on control plants (Fig. 3A).

A separate group of plants were inoculated with 15 2-day-old nymphs. These nymphs were allowed to feed for 19 d, starting on plants at 7 dpni, to assess aphid population growth (Fig. 3B). Despite Pp-infected plants supporting the smallest aphid populations, the overall model shows that nematode treatments did not significantly affect aphid population growth at this time point (*F*_3,35_=1.077, *P*=0.372).

Overall, aphid survival rates at 3 dpai were significantly lower on plants pre-infected with *P. penetrans* or those with MP treatment, while nematode treatments did not significantly affect aphid population growth after 19 d.

### Nematodes systemically altered leaf phytohormone and glucosinolate concentrations

#### Phytohormones

**Fig. 4.**
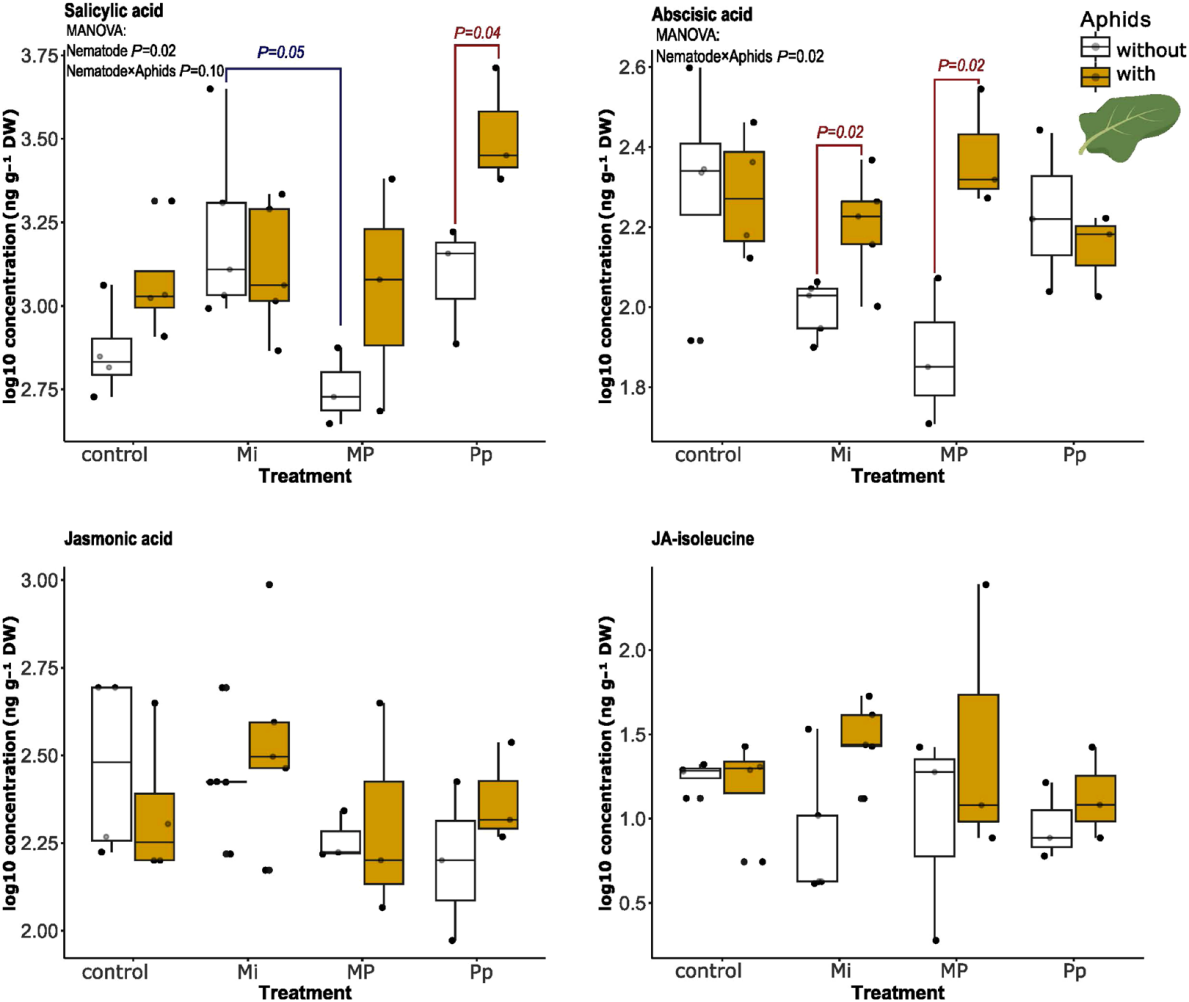
Log_10_-scaled concentrations of the phytohormones salicylic acid (SA), abscisic acid (ABA), jasmonic acid (JA), and the conjugate JA-Ile extracted from the leaves of *Brassica nigra* plants infected with either individual or combinations of nematodes [*Meloidogyne incognita* (Mi), *Pratylenchus penetrans* (Pp), and *M. incognita* and *P. penetrans* together (MP)] and aphids (*Brevicoryne brassicae*) at 3 d after aphid infestation. Connected boxplots are significantly different based on specific significant predictors in the MANOVA model: blue, nematode effect; red, nematode×aphid effect. Pairwise comparison of statistically significant factors from the model was assessed via pairwise *t*-tests at *P*<0.05. Points represent the number of biological replicates per treatment (control, *n*=4; Mi, *n*=5; MP and Pp, *n*=3).

Root infection with PPNs systemically affected the measured phytohormone profile in the leaves as indicated by MANOVA results (Fig. 4; Pillai’s trace: *F*_3,63_=3.212, *P*=0.002). This was mostly driven by the overall effect of nematode treatments on SA concentrations (ANOVA: *F*_3,22_=4.94, *P*=0.009). The SA concentrations in leaves of Mi-infected plants were overall higher than in the leaves of MP-treated plants (Fig. 4, upper-left; pairwise *t*-test: *P*_adj_=0.05). Leaf SA concentrations in MP-treated plants without aphids were not significantly different from those of Pp-treated plants (pairwise *t*-test: *P*_adj_=0.38).

Nematode treatment also affected aphid-induced phytohormone concentrations (MANOVA; Pillai’s trace: aphid×nematode *F*_3,63_=2.35, *P*=0.015). This interaction effect was statistically significant for leaf ABA concentrations (ANOVA: *F*_3,22_=3.97, *P*=0.015) and marginally for SA concentrations (ANOVA: *F*_3,22_=2.33, *P*=0.102). In Mi- (pairwise *t*-test: *P*_adj_=0.02) and MP- (pairwise *t*-test: *P*_adj_=0.02) infected plants, aphid feeding caused significant accumulation of ABA in the leaves compared with the corresponding plants with nematodes only (Fig. 4, upper right). The presence of aphids in Pp-infected plants caused a significant increase in leaf SA concentrations (pairwise *t*-test: *P*_adj_=0.04) (Fig. 4, upper left). The levels of JA and JA-Ile did not change in response to either nematode or aphid infections. A complete MANOVA table is given in Supplementary Table S2.

#### Glucosinolates

**Fig. 5.**
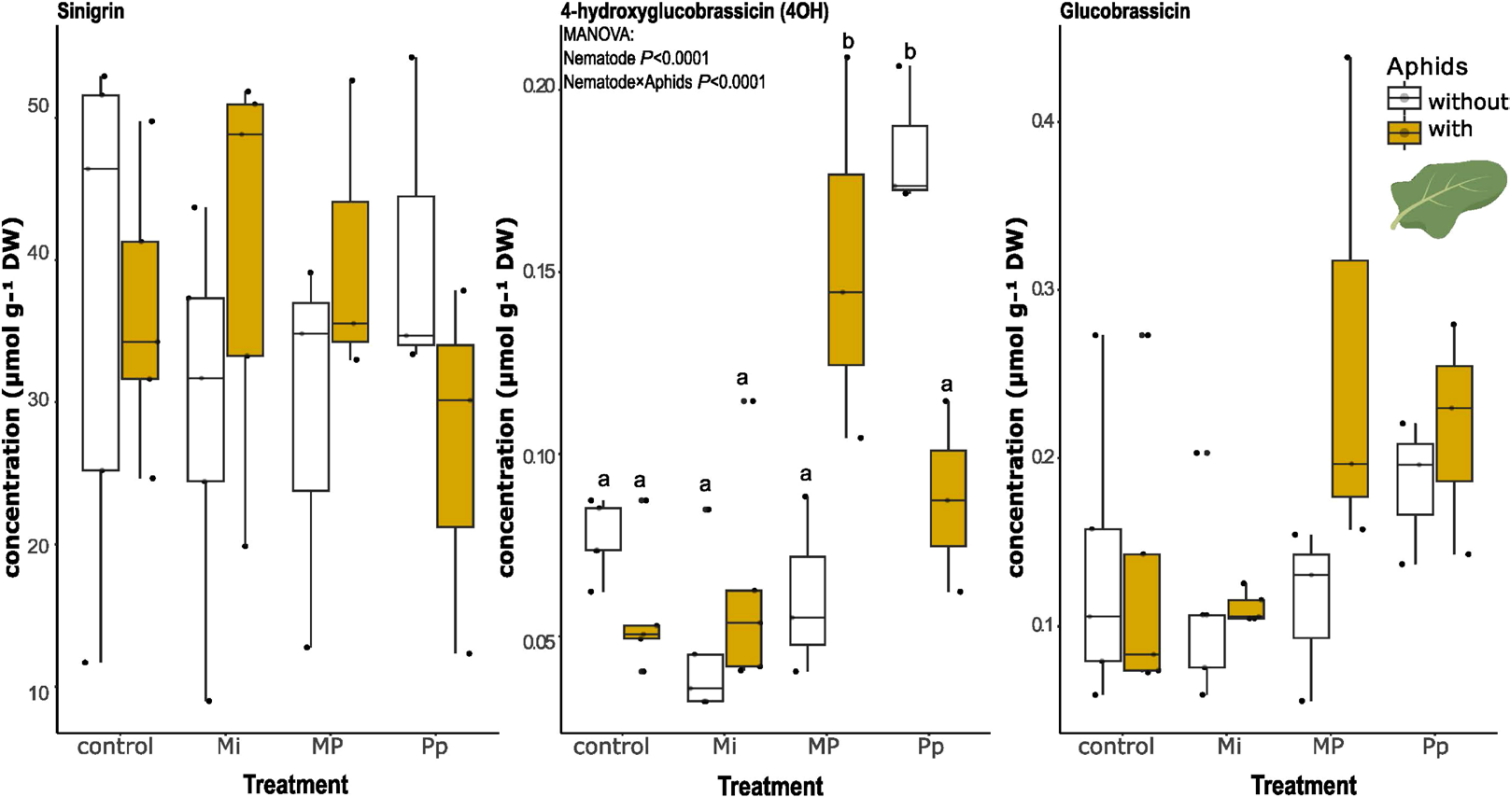
Aliphatic (sinigrin) and indole (4-hydroxyglucobrassicin and glucobrassicin) glucosinolate concentrations in *Brassica nigra* leaves from plants infected with either individual or combinations of nematodes [*Meloidogyne incognita* (Mi), *Pratylenchus penetrans* (Pp), and *M. incognita* and *P. penetrans* together (MP)] and aphids (*Brevicoryne brassicae*) 3 d after aphid infestation, as measured on HPLC–photodiode array detector. Boxplots labeled with different letters are significantly different as per the pairwise *t*-test with Bonferroni *P*-adjustment at *P*<0.05. Points represent the number of biological replicates per treatment (Mi and control, *n*=5; MP and Pp, *n*=3).

Nematode treatments (MANOVA; Pillai’s trace: *F*_3,72_=0.77, *p*=0.008) and the interaction between nematode and aphids (MANOVA; Pillai’s trace: *F*_3,72_=3.25, *P*=0.002) significantly affected leaf glucosinolate concentrations (Fig. 5), particularly the indole glucosinolate, 4-hydroxyglucobrassicin (Fig. 5, middle; ANOVA_nematode_: *F*_3,24_=15.4, *P*<0.001; ANOVA_nematode×aphid_: *F*_3,24_=13.7, *P*<0.001). Before aphid infection, 4-hydroxyglucobrassicin concentrations in Pp-infected plants were significantly higher than in control plants (pairwise *t*-test: *P*_adj_<0.001) or Mi- (pairwise *t*-test: *P*_adj_<0.001) and MP- (pairwise *t*-test: *P*_adj_<0.001) treated plants. Aphid feeding on MP-treated plants caused a significant increase in leaf 4-hydroxyglucobrassicin concentrations compared with MP plants without aphids (pairwise *t*-test: *P*_adj_<0.01). Aphid feeding on Pp-infected plants resulted in a significant reduction of 4-hydroxyglucobrassicin (pairwise *t*-test: *P*_adj_<0.01). The leaf concentration of sinigrin, the most abundant glucosinolate in *B. nigra*, was not affected by any of the treatments. The results for total indole glucosinolates, that is the sum of 4-hydroxyglucobrassicin and glucobrassicin, are shown in Supplementary Fig. S6. The full MANOVA table is given in Supplementary Table S3.

These targeted analyses showed that *M. incognita* and *P. penetrans* alone had modest systemic effect on leaf phytohormone and GSL concentrations. Aphid feeding further modulated these changes, significantly increasing ABA in Mi- and MP-treated plants, and SA levels in Pp-treated plants compared with their nematode-infected counterparts. Aphid infection also altered GSL profiles, particularly by decreasing 4-hydroxyglucobrassicin in Pp-treated plants, while increasing its levels in MP-treated plants compared with their nematode-infected counterparts.

### Species- and time-dependent effect of herbivores on phloem metabolites

**Fig. 6.**
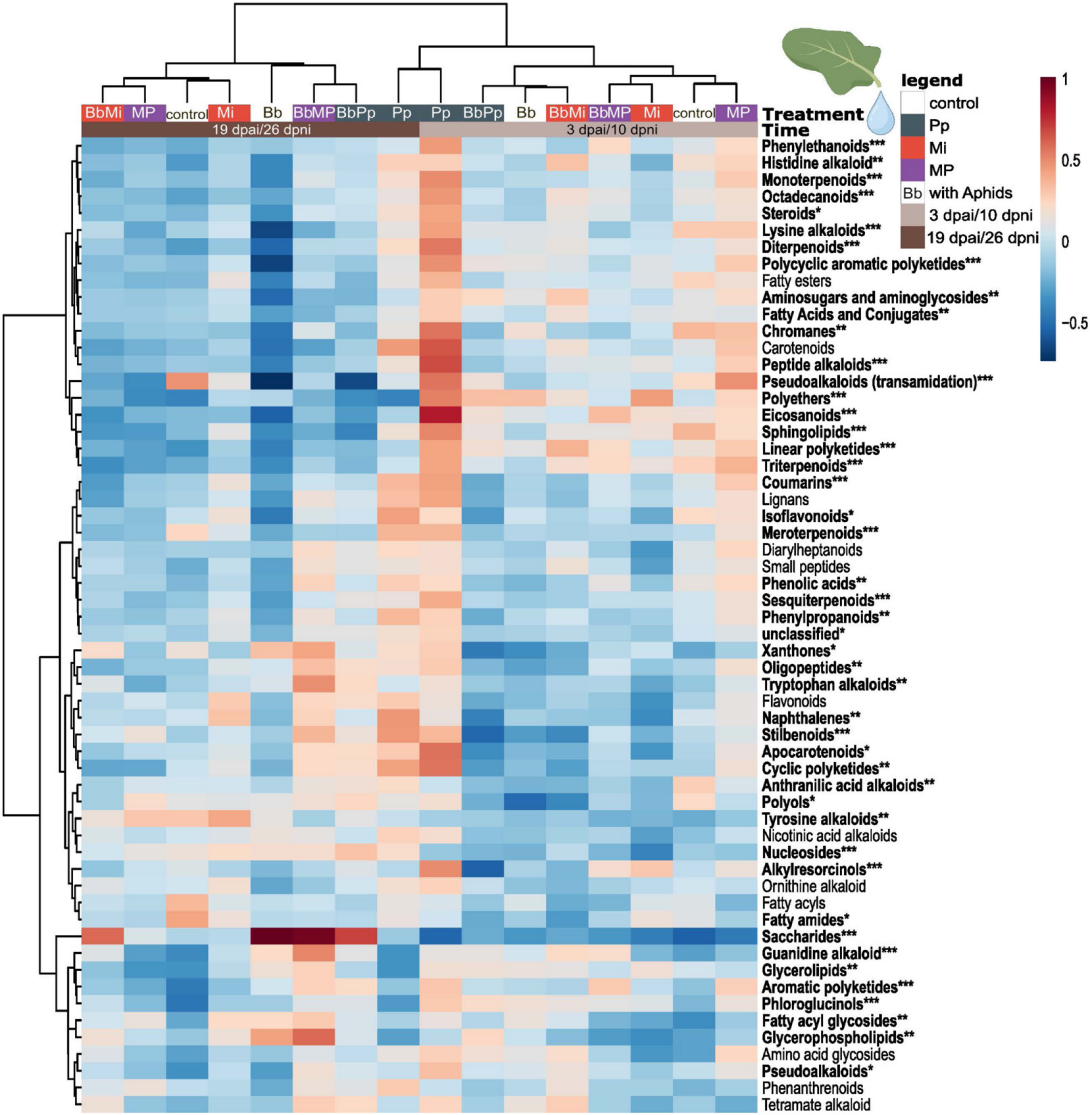
Heatmap showing clustering result of features grouped according to NPClassifier superclass from untargeted phloem analysis of plants treated with individual nematodes [*Meloidogyne incognita* (Mi), *Pratylenchus penetrans* (Pp)], simultaneous nematode infection [*M. incognita* and *P. penetrans* together (MP)], aphids [*Brevicoryne brassicae* (Bb)], and successive nematode–aphid infection at early (10 dpni, 3 dpai) or later (26 dpni, 19 dpai) time points. Clustering was based on Euclidean distance and Ward algorithm methods. Red and blue colors represent the relative abundance of compounds based on log-transformed and Pareto-scaled peak intensities, shown as the average of 10 biological replicates per treatment; red indicates higher relative intensity, blue indicates lower relative intensity. dpai, days post-aphid inoculation; dpni, days post-nematode inoculation.

In addition to the important features in the phloem shown by PLS-DA VIP analysis, we found specific compound groups associated with the interaction between nematodes and aphids. Using the superclass classification via NPClassifier, we performed PERMANOVA to assess differences in phloem metabolite composition between nematode- and aphid-infested plants based on the features grouped at superclass level. We also included the effect of either early (3 dpai, 10 dpni) or later (19 dpai, 26 dpni) infection time points in the model. Results point to nematode treatments explaining 5.41% of the total variation. Aphids (1.98%) and data-collection time points (day, 2.54%) also significantly explained the total variation. The interaction of these three factors explained 3.74% of the total variation (Supplementary Table S4). To illustrate these results, we show a heatmap denoting relative abundances of each compound group based on peak intensities (Fig. 6). The statistically significant groups are listed in Supplementary Table S4. Notably, several phenolic groups were up-regulated in the phloem of Pp-infected plants at 10 dpni (Fig. 6). Saccharides on the other hand, were increased by aphid infection at the later time point (Fig. 6).

**Fig. 7.**
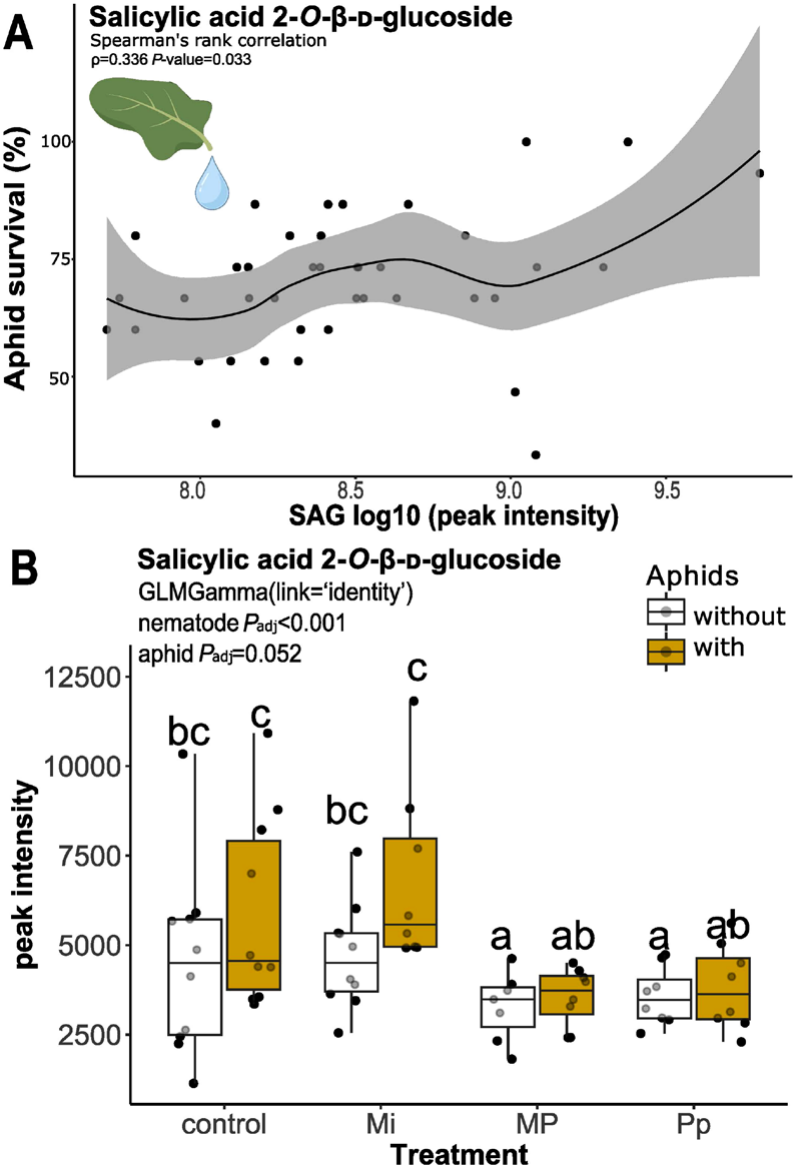
Salicylic acid-2-*O*-β-d-glucoside (SAG) in the phloem of nematode [*Meloidogyne incognita* (Mi), *Pratylenchus penetrans* (Pp), and *M. incognita* and *P. penetrans* together (MP)] and aphid-infected *Brassica nigra* plants. (A) Scatterplot showing the significant positive relationship between SAG peak intensity and aphid survival at 3 dpai. The correlation coefficient (ρ) was estimated by Spearman’s rank correlation test. The black dots represent the data points. The black line represents a smooth spline fit with a gray ribbon representing the confidence interval. (B) Boxplots showing log-scaled peak intensity of SAG. Boxplots labeled with different letters are significantly different as per the pairwise test of estimated marginal means (emmeans) at *P*<0.05. Points represent the number of biological replicates per treatment (Mi and Control, *n*=10; MP and Pp, *n*=8). dpai, days post-aphid inoculation.

We further investigated whether any annotated features in the phenolic groups were associated with aphid survival and population growth. Using Spearman’s rank correlation test, we found that salicylic acid 2-*O*-β-d-glucoside (SAG) had a significant positive relationship with aphid survival at 3 dpai (*S*=7072.4, ρ=0.34, *P*=0.033) (Fig. 7A). Generalized linear model showed that nematodes (GLM: χ^2^=19.199, df=3, *P*<0.001) significantly affected SAG levels in the phloem. Aphids (GLM: χ^2^=3.776, df=1, *P*=0.052) marginally affected phloem SAG levels. Pairwise comparisons showed that the mean phloem SAG peak intensity in Mi-treated plants was significantly higher than in MP- [estimated marginal means (emmeans) *P*_adj_=0.004] and Pp- (emmeans *P*_adj_=0.010) treated plants. MP- (emmeans *P*_adj_=0.009) and Pp- (emmeans *P*_adj_=0.032) treated plants also had significantly lower SAG levels compared with control plants. The SAG levels in aphid–Mi infected plants were also significantly higher than in aphid–MP (emmeans *P*_adj_=0.004) and aphid–Pp (emmeans *P*_adj_=0.010) infected plants (Fig. 7B).

These results showed that the systemic effects of *M. incognita* and *P. penetrans* on the metabolic profile of *B. nigra* phloem varied between early and later nematode infestation and aphid feeding time points. In particular, Pp and MP treatments resulted in a significant increase in phenolic compounds at 10 dpni. Among the phenolic compounds, SAG levels in the phloem were significantly reduced by Pp and MP treatments. The SAG levels in the phloem were positively correlated with aphid survival, indicating that low SAG levels may potentially be associated with reduced aphid survival.

### Influence of nematodes and aphids on phloem sugars

**Fig. 8.**
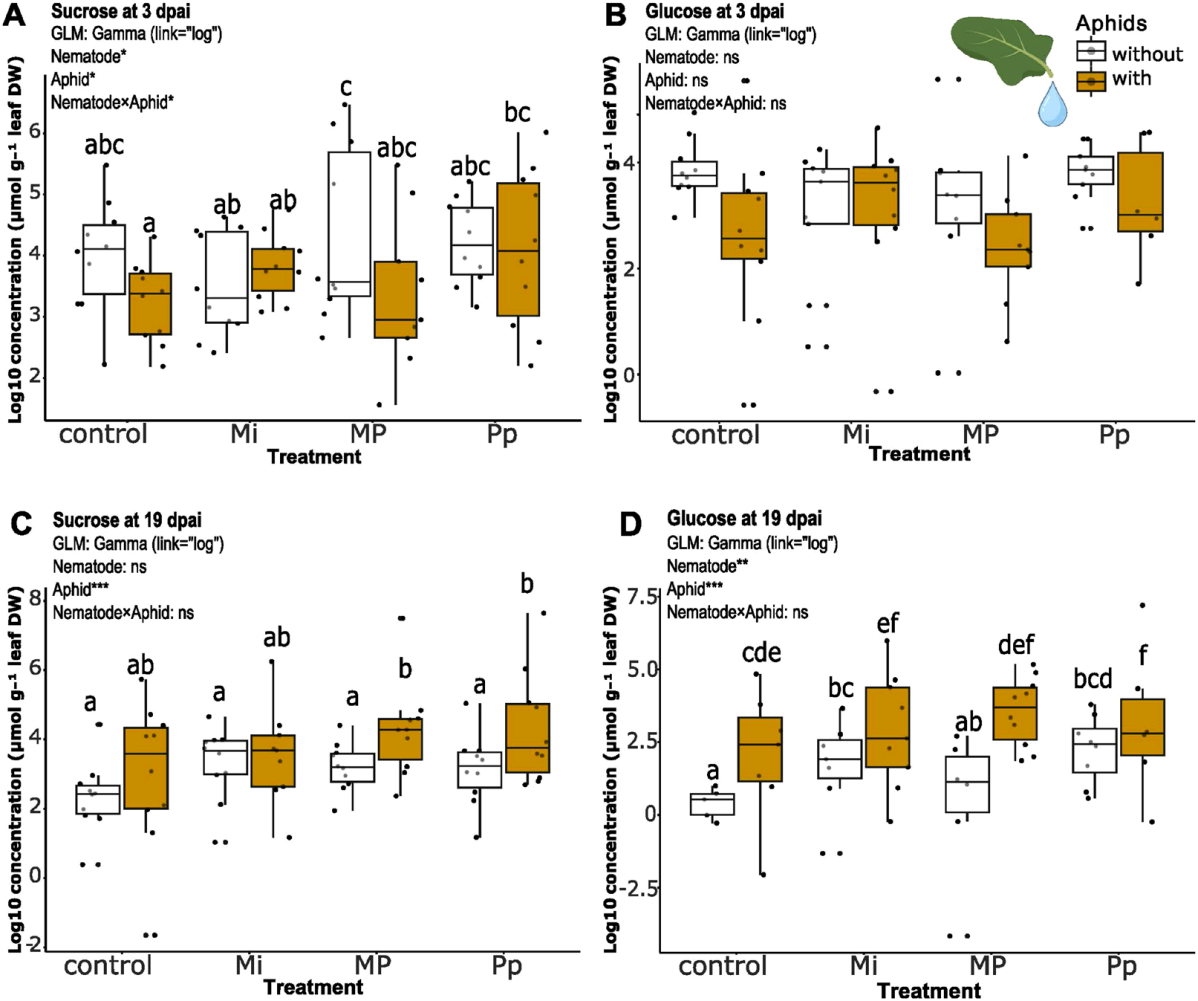
Log_10_-scaled concentrations of glucose (B, D) and sucrose (A, C) in *Brassica nigra* phloem from plants infected with either individual, or combinations of nematodes [*Meloidogyne incognita* (Mi), *Pratylenchus penetrans* (Pp), and *M. incognita* and *P. penetrans* together (MP)] and aphids (*Brevicoryne brassicae*) at 3 d (A, B) and 19 d (C, D) after aphid infestation as measured on HPLC–charged aerosol detector. Boxplots labelled with different letters are significantly different as per pairwise test via estimated marginal means (emmeans) at *P*<0.05. Points represent the number of biological replicates per treatment (*n*=5–10). dpai, days post-aphid inoculation.

To determine the influence of belowground nematode infections on quantifiable sugars present in the phloem samples, we analysed the changes in sucrose and glucose concentrations over the early (3 dpai) and later (19 dpai) herbivore feeding time points. At 3 dpai, phloem sucrose concentrations (Fig. 8A) were affected by nematodes (GLM: χ^2^=11.187, df=3, *P*=0.01), aphids (GLM: χ^2^=4.558, df=1, *P*<0.05), and the interaction between nematodes and aphids (GLM: χ^2^=8.486, df=3, *P*<0.05), while phloem glucose concentrations (Fig. 8B) were not significantly affected by either of the herbivores, nor their interaction. At this time point, MP infection caused a systemic increase of sucrose concentration in the phloem compared with that of Mi-treated plants (*P*=0.021), yet this increase in sucrose concentration was not significantly different from that of control and Pp-treated plants. Aphids generally reduced phloem sucrose concentrations at 3 dpai. The combined infection of Pp and aphids significantly increased sucrose concentration compared with that of plants infected with aphids alone (*P*=0.0215).

The results obtained at 19 dpai showed that the general increase of sucrose (Fig. 8C) concentrations in the phloem can be linked to aphid infection (GLM: χ^2^=17.650, df=1, *P*<0.001). The aphid-induced increase of sucrose concentrations was more pronounced in aphid-infested plants with prior MP and Pp infection (Fig. 8C). The phloem sugar concentration in these groups differed significantly from that of nematode infested plants without aphids (*P*<0.05) as well as from the control group (*P*<0.01). Glucose concentrations, on the other hand (Fig. 8D), were influenced by nematodes (GLM: χ^2^=14.937, df=3, *P*<0.01) and aphids (GLM: χ^2^=34.132, df=1, *P*<0.001) independently. Mi and Pp treatments resulted in increased phloem glucose concentrations compared with the control group (*P*<0.05). The mean phloem glucose concentration in plants with aphids and Pp were significantly higher than the control group (*P*<0.001) and higher than its single-species counterparts (aphid only: *P*<0.05; Pp only: *P*<0.01).

In general, sucrose and glucose levels in the phloem increased mostly due to aphid feeding at the later time point. At the same time, phloem glucose concentrations increased due to nematode infestation, especially by Pp infection.

## Discussion

Using metabolomic analyses, we show that single-species infections by *M. incognita* and *P. penetrans* in the roots led to discrete metabolic changes in the leaves and phloem of *B. nigra*. Other than postulated, that simultaneous nematode infection will elicit an attenuated defense response, our results show that concurrent infection elicited responses that differ from single-species infections (Fig. 2). Much of the MP-induced phloem metabolic profile changes suggested that the responses elicited by either species dominate at certain infection time points. These observations matched with the effect of MP infection on the performance of aphids. Specifically, aphid survival was reduced on MP-treated plants at the early infection stage (3 dpai, 10 dpni), which was similar to what we observed on Pp-treated plants (Fig. 3A). At the later aphid feeding time point (19 dpai, 26 dpni), the effect of MP infection on the phloem metabolome resembled that of Mi-induced effects (Fig. 6). By comprehensively analysing leaf and phloem metabolomes, we showed that systemic-induced plant responses can potentially mediate these aboveground–belowground herbivore interactions. While studies on one herbivore–one plant systems provide important information on molecular mechanisms underlying plant-mediated interactions between root and shoot phytophages, our results showed that concurrent nematode infections may change such plant-mediated interactions, potentially via changes in aboveground plant metabolomes that may affect aboveground herbivores. Because concurrent infections by different root feeding nematode species are common in natural environments, our study contributes to a better understanding of how plant-mediated interactions may shape induced plant responses to aboveground feeding herbivores in nature.

### Plant shows discrete metabolic responses to plant-parasitic nematodes of different feeding strategies

Our results showed that *M. incognita* and *P. penetrans* infections systemically induced changes in *B. nigra* leaf and phloem metabolic profiles (Figs 2, 6). *Pratylenchus penetrans* infection up- and down-regulated more (unique) features than *M. incognita* (Fig. 2E, F, K, L). These distinct plant responses to *M. incognita* and *P. penetrans* infections may be attributed to the different feeding strategies and effectors employed by the two PPN species (Lohmann *et al.*, 2009; Hol *et al.*, 2016; van Dam *et al.*, 2018). The stronger response to *P. penetrans* infection is understandable when considering that these nematodes are mobile and continuously damaging root cells. In contrast, *M. incognita* is sedentary and actively manipulates the plant to create a sink and suppress immune responses (Gheysen and Mitchum, 2011; Jones *et al.*, 2013). This difference was also shown in other plant systems (Lohmann *et al.*, 2009; Kyndt *et al.*, 2012b). In rice, Kyndt *et al.* (2012b) reported that root infection by the migratory endoparasitic nematode *H. oryzae* systemically induced several defense-related genes in the shoots at an early infection stage, while the same genes were suppressed in the shoots of plants infected with the sedentary endoparasitic nematode *M. graminicola*. Our findings indicate that there are more DA features in the leaves and phloem of *B. nigra* in response to root infection by the migratory nematode *P. penetrans* than to infection by the sedentary endoparasitic nematode *M. incognita* at 10 dpni (Fig. 2E, F, K, L). Plant metabolic changes differentiating the effects of necrotrophic versus biotrophic pathogens (Glazebrook, 2005; McCombe *et al.*, 2022) or that of leaf-chewing versus phloem-sucking herbivores (Walling, 2000; Kaloshian and Walling, 2005; Davidson-Lowe and Ali, 2021) suggest that activation of inducible plant responses to chewing herbivores or necrotrophic pathogens is faster than those to biotrophic pathogens or aphids (De Vos *et al.*, 2005; Kloth and Dicke, 2022; Musaqaf *et al.*, 2023). This was attributed to the immediate and relatively high damage that leaf chewers and necrotrophic pathogens cause to plant tissues, while phloem feeders and biotrophic pathogens avoid plant damage and suppresses plant defense (Walling, 2000; De Vos *et al.*, 2005; Kaloshian and Walling, 2005; Musaqaf *et al.*, 2023). Similar differences in response timing were found for migratory and sedentary endoparasitic nematodes, for both local and systemic plant defense responses (Kyndt *et al.*, 2012a, b; van Dam *et al.*, 2018).

In parallel with the discrete changes in shoot metabolome profiles in response to *M. incognita* and *P. penetrans*, we found differences in the survival of aphids feeding on these nematode-infested plants (Fig. 3). Our results align with the previous report of Hol *et al.* (2016), where the number of aphids on *M. incognita*-infected plants was slightly higher than the number of aphids on the control plants, even though not statistically significant. When plants were infected with the related species, *Meloidogyne hapla*, significantly higher number of aphids were found on these plants, when compared with control plants (van Dam *et al.*, 2018). This may suggest that while root-knot nematodes tend to positively affect aphid performance, the effects may be nematode species-specific. In the phloem of *M. incognita*-infected plants, a significant reduction of features characterized as flavonoids was observed, including a feature putatively annotated as luteolin (Supplementary Fig. S5). Luteolin was reported to negatively affect the feeding behavior of the aphid, *Acyrthosiphon pisum* when spiked in its artificial diet (Goławska and Łukasik, 2012). In this context, the *M. incognita*-induced reduction of flavonoids in the current experiment may have provided a conducive environment for aphid performance. This may explain the slight positive effect of belowground *M. incognita* infection on aphid survival and population growth in our experiments, which corroborates previous studies using similar set-ups (Hol *et al.*, 2016; van Dam *et al.*, 2018). Further bioassays that will look into aphid performance on mutant plants with altered levels of luteolin should be performed to validate these observations.

*Pratylenchus penetrans-*infected plants held a significantly lower number of surviving aphids compared with the control and *M. incognita*-infected plants (Hol *et al.*, 2016). Our results show that leaf metabolic changes in response to *P. penetrans* at 10 dpni involve the accumulation of l-tryptophan (Supplementary Figs S3, S7), an essential amino acid and the precursor of indole glucosinolates (Hull *et al.*, 2000; Kim and Jander, 2007; Bednarek *et al.*, 2011). In particular, 4-hydroxyglucobrassicin levels increased in *P. penetrans*-infected plants. Studies showed that 4-hydroxyglucobrassicin may confer resistance to aphids and pathogens (Kim and Jander, 2007; Kuśnierczyk et al., 2008; Clay *et al.*, 2009). While differential accumulation of specific indole glucosinolates and breakdown products coincided with the reduction in aphid survival on *P. penetrans-*infected plants, a causal link has yet to be established. Targeted measurements of indole glucosinolate breakdown products and bioassays in aphids would be needed to corroborate our findings.

*Pratylenchus penetrans* infection systemically reduced the accumulation of ascorbic acid in the leaves (Supplementary Fig. S3). This may be explained by the role of ascorbic acid as an antioxidant (Kerchev *et al.*, 2013). Increased root damage caused by *P. penetrans* migration and feeding possibly drove the plant to redirect this antioxidant resource to belowground tissues, which may have reduced its availability aboveground. The early *P. penetrans* infection was also characterized by increased accumulation of leaf syringic and sinapic acids (Supplementary Fig. S3). Both are hydroxycinnamic acids derived from downstream processes in the phenylalanine ammonia-lyase pathway, where many defense-associated compounds are produced (Chrzanowski *et al.*, 2012; Florencio-Ortiz *et al.*, 2021). Indeed, syringic and sinapic acids were reported to negatively affect herbivores, including aphids (Usha Rani and Pratyusha, 2014; Florencio-Ortiz *et al.*, 2021; Gebretssidak *et al.*, 2022). To our knowledge, these compounds have not yet been tested on *B. brassicae*.

We also compared the early herbivore-induced changes (3 dpai, 10 dpni) in phloem with the changes after a prolonged herbivore-feeding period (19 dpai, 26 dpni). Our results showed that while nematode infection still affected the phloem metabolome (Fig. 6; Supplementary Table S4), most of the changes were associated with aphid feeding, especially the enhanced level of saccharides (Figs 6, 8C, D; Supplementary Table S4). Aphids inject effectors to create a sink to their feeding site, which may also enhance the levels of saccharides (sugars) in the phloem (Züst and Agrawal, 2016; Jakobs *et al.*, 2019). Our data show, however, that the aphids’ ability to manipulate the phloem sap (Jakobs *et al.*, 2019) was not affected by the presence of nematodes.

This may explain why aphid populations in the end were not significantly impacted in the presence of nematode infections.

### Plant response to concurrent nematode infection differed from single species infections

While it is common knowledge that plants are attacked by several species of PPN at the same time (Estores and Chen, 1972; Fontana *et al.*, 2015; Hol *et al.*, 2016), studies on plant response towards simultaneous PPN infections are scarce. Our results suggest that concurrent infection by *M. incognita* and *P. penetrans* induced changes in the leaf and phloem metabolic profiles that were different from those induced by single PPN infections (Fig. 2). This is evident in the high number of leaf and phloem DA metabolites that are unique to MP infection. On other plant species, *Meloidogyne* spp. and *Pratylenchus* spp. were documented to have antagonistic relationships (Gay and Bird, 1973; Chapman and Turner, 1975; Fontana *et al.*, 2015). Although our experiments did not allow for the evaluation of nematode population increase and density-dependent species dominance over a longer time period, the plant’s response, as reflected by the metabolic profile, provided novel insights. At the early infection stage, the leaf and phloem metabolic profiles of MP-infected plants share more DA metabolites with *P. penetrans*- than with *M. incognita*-infected plants (Fig. 2E, F, K, L). This time point-dependent dominance of *P. penetrans*-induced responses is also reflected in the effect of MP treatment on aphid survival. We interpret these data such that *P. penetrans* triggers stronger defense responses than *M. incognita* early in the infection process, because of being a migratory endoparasite. In contrast, *Meloidogyne* spp. are reported to suppress plant immune responses early in the infection process, enabling them to establish permanent feeding sites (Abad and Williamson, 2010; Mitchum *et al.*, 2013). The similarity of plant metabolic profiles of MP- and *P. penetrans* -treated plants at 10 dpni suggests that the plant’s response to *P. penetrans* is stronger than the suppression by *M. incognita*. At 26 dpni, the pattern of feature peak intensities in the phloem metabolome of MP-infected plants resembled that of *M. incognita*-infected plants rather than that of *P. penetrans*-infected plants (Fig. 6). This follows the similarity of MP-induced aphid population growth response to that of *M. incognita*-infected plants. This is in line with results obtained by Mbaluto *et al.* (2020), who showed that at 30 d post-*M. incognita* inoculation the increase in tomato root defense compounds was stronger than at early infection stages. These time-dependent differences may also explain the seemingly dominant effect of *M. incognita* at 26 dpni on the plant response in MP plants. The observed similarities between MP and *P. penetrans* at 10 dpni and the resemblance of MP- and *M. incognita*-infected phloem metabolic profiles at 26 dpni may suggest a change in species dominance over time. Future studies that can track nematode populations and their interactions over longer time periods could provide additional insights into the role of nematode population dynamics.

### Phytohormone-mediated interactions between nematodes and aphids

The notion that PPNs with different feeding strategies discretely affect aphid performance aboveground was previously attributed to the differences in phytohormonal pathways involved in response to each nematode species (Wondafrash *et al.*, 2013; van Dam *et al.*, 2018). Our results show that *P. penetrans* infection at 10 dpni is characterized by enhanced accumulation of the JA precursors, linoleic acid and OPDA, in the leaves (Supplementary Fig. S3). However, there were no apparent changes in JA and JA-Ile levels that could be associated to any of the nematode treatments (Fig. 4). Linoleic acid is a polyunsaturated fatty acid from which α-linolenic acid is synthesized (Wallis *et al.*, 2002; Li *et al.*, 2016). α-Linolenic acid is the precursor of the JA pathway, which after a series of enzymatic reactions is converted to OPDA (León and Sánchez-Serrano, 1999; Li *et al.*, 2016). The increase of linoleic acid and OPDA may contribute to the synthesis of JA and other oxylipins involved in plant defense (León and Sánchez-Serrano, 1999; Li *et al.*, 2016). Without apparent increase in JA and Ja-Ile at the times we sampled, additional experiments, for example using qPCR analyses of JA-regulated genes, would be needed to conclusively assess whether *P. penetrans* feeding triggers the JA pathway.

In the phloem, *P. penetrans* induced the significant reduction of SAG (Fig. 7). On one hand, SAG is considered as a storage form of SA that can be hydrolysed to release active SA when needed (Ratzinger *et al.*, 2009; Umemura *et al.*, 2009; Bonnemain *et al.*, 2013). In addition, SAG may be the form in which SA is transported throughout the plant, which would explain its presence in the phloem. This transport may be essential for the induction of systemic acquired resistance that enhances the ability of plants to resist future pathogen attacks (Walters and Heil, 2007; Umemura *et al.*, 2009; Bonnemain *et al.*, 2013). On the other hand, the reduced SAG levels may also indicate an altered SA metabolism, which may result to changes in the availability of free SA for defense (George Thompson *et al.*, 2017; Kobayashi *et al.*, 2020). For example, Arabidopsis plants with knocked-out *UGT74F2* (*ugt74f2* mutant), a gene coding for a UDP-glucosyltransferase enzyme that catalyses SA glycosylation to SAG and SA glucose ester, show higher SA levels and increased resistance to *Pseudomonas syringae* (Dean and Delaney, 2008; Boachon *et al.,* 2014; George Thompson *et al.*, 2017). Free SA also serves as a direct defense against aphids (Donovan *et al.*, 2013). Increased SA levels were reported to deter *B. brassicae* feeding or negatively impact their reproduction (Khoshfarman-Borji *et al.*, 2020; Rubil *et al.*, 2022). Given the limitations of our study, we could not assess whether the observed *P. penetrans*-induced reduction of phloem SAG (Fig. 7) led to the increase in free SA. To date, the details on SAG manipulation in the context of plant–aphid interactions are not yet clearly defined, thereby forming an interesting prospect for future research.

Our results also show high levels of ABA in the phloem of *P. penetrans*-infected plants (Supplementary Fig. S8). ABA is often associated with responses to abiotic stress, such as drought and high salinity. Altered ABA levels may lead to changes in the nutritional quality of phloem sap (Guo *et al.*, 2016). Dehghan *et al.* (2023) reported that ABA induces a detrimental effect on the development and survival of *B. brassicae* nymphs and correlated these effects to increased phenolics. Our results concurred with these observations, as we also found significantly higher levels of coumarins and phenylpropanoids, and marginally higher levels of flavonoids in the phloem metabolome of Pp-infected plants (Fig. 6; Supplementary Table S4). These *P. penetrans-*induced changes correlated with reduced aphid survival in *P. penetrans*-infected plants. Further targeted bioassays to assess the effect of ABA and changes in the phenylpropanoid levels on *B. brassicae* are needed.

We found that phytohormone-induced responses to MP infections were not attenuated (Figs 4, 7; Supplementary Fig. S8), meaning that the different phytohormonal responses induced by each nematode are not cancelling each other out. For instance, the contrasting effects of *M. incognita* and *P. penetrans* on phloem SAG and ABA levels did not result in intermediate levels in MP-treated plants. Instead, SAG and ABA levels in MP-treated plants follow the same pattern as in *P. penetrans-*infected plants, consistent with a putative dominance of the *P. penetrans*-induced response at this time point.

### Aphid feeding alters phloem sugars and interaction with nematodes influences dynamics

Aphids primarily feed on the phloem, where they take up nutrients, such as sugars and amino acids (Züst and Agrawal, 2016; Jakobs *et al.*, 2019; Chen *et al.*, 2020). Aphids also inject effectors to create an active sink at their feeding site and to suppress plant responses that may lead to phloem blockage (Züst and Agrawal, 2016). Our results show that changes in phloem sucrose and glucose concentrations are particularly affected by aphids (Fig. 8). Sucrose is the sugar possessing the greatest nutritional value for aphids and is favored over other sugars by certain species (Hewer *et al.*, 2010; Jakobs *et al.*, 2019). The higher glucose concentrations in plants infected with both aphids and *P. penetrans* compared with single-species treatments may suggest a potential synergistic effect on phloem glucose levels. Alternatively, the glucose we found may be a consequence of the employed technique. As pointed out by Broussard *et al.* (2023), sucrose-cleaving enzymes active during the exudation process affect the results from this method. Further research is needed to support the results obtained herewith. We suggest looking into the expression of genes related to sugar metabolism and transport in response to nematode and aphid infections.

Taken together, our study reveals distinct metabolic responses in plants when challenged by PPNs with different feeding strategies. The use of untargeted metabolomics further showed the modulation of aboveground plant responses to PPN infections and the possible effects on aphid survival. This study provides novel evidence that concurrent infections by *M. incognita* and *P. penetrans*, as can occur in natural environments, elicited responses that differ from single-species infections, indicating a distinct plant response to concurrent infection. *Brassica nigra* is an economically important crop and a genetic repository of many commercially grown vegetables, and this work will be of particular importance to address challenges on the presence of multiple pests affecting related crops.

## Supplementary data

The following supplementary data are available at *JXB* online.

Fig. S1. Nematode infection confirmation.

Fig. S2. Top 10 and top 50 variable importance in projection (VIP) on the first component after partial least squares discriminant analysis (PLS-DA) of the untargeted leaf metabolome data at 10 dpni.

Fig. S3. Boxplots of differentially abundant features in the leaves of nematode-infected (Mi, MP, Pp) plants versus uninfected control plants.

Fig. S4. Top 10 and top 50 variable importance in projection (VIP) on the first component after partial least squares discriminant analysis (PLS-DA) of the untargeted phloem metabolome data at 10 dpni.

Fig. S5. Boxplots of differentially abundant features in the phloem of nematode-infected (Mi, MP, Pp) plants versus uninfected control plants.

Fig. S6. Total indole glucosinolate concentrations in *Brassica nigra* leaves from plants infected with either individual or combinations of nematodes and aphids.

Fig. S7. l-Tryptophan peak intensities from untargeted data of *Brassica nigra* leaves from plants infected with either individual or combinations of nematodes and aphids.

Fig. S8. Abscisic acid (ABA) peak intensities from untargeted data of *Brassica nigra* phloem from plants infected with either individual or combinations of nematodes and aphids.

Table S1. Classified leaf and phloem feature tables and list of differentially abundant leaf and phloem metabolites (separate excel file).

Table S2. MANOVA table for leaf phytohormones.

Table S3. MANOVA table for leaf glucosinolates.

Table S4 Results of PERMANOVA and univariate analyses of compound superclass from the untargeted phloem metabolome data.

erae364_suppl_Supplementary_Figures_S1-S8_Tables_S2-S4

erae364_suppl_Supplementary_Table_S1

## Data Availability

The data used in this paper are available in Zenodo (https://doi.org/10.5281/zenodo.12528317) (Pajar *et al*., 2024).
