# Supplementary material for: Dual nematode infection in Brassica nigra affects shoot metabolome and aphid survival in distinct contrast to single-species infection
Source: J Exp Bot. 2024 Aug 29;75(22):7317–36. doi: 10.1093/jxb/erae364 (PMC11630020; doi:10.1093/jxb/erae364)
Supplement: erae364_suppl_Supplementary_Figures_S1-S8_Tables_S2-S4 [file erae364_suppl_supplementary_figures_s1-s8_tables_s2-s4.pdf]

Dual nematode infection in *Brassica nigra* affects shoot metabolome and aphid survival in distinct contrast to single-species infection

Jessil Ann Pajar, Pius Otto, April Lyn Leonar, Stefanie Döll, and Nicole M. van Dam

Supplementary information:

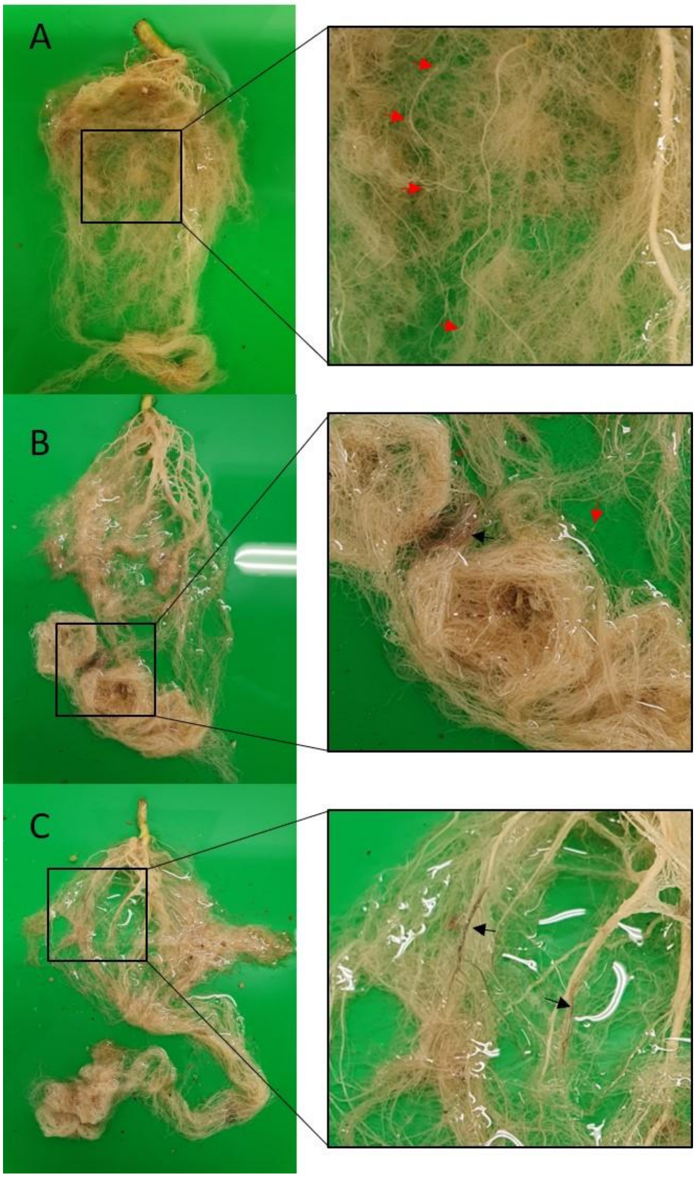

Fig. S1 Photos confirming nematode-infection. *Brassica nigra* roots infected with (A) *Meloidogyne incognita* only, (B) *Meloidogyne incognita* + *Pratylenchus penetrans*, and (C) *Pratylenchus penetrans* only. Red arrows are pointed to root galls, black arrows point to root lesions—indicating *M. incognita* and *P. penetrans* infections, respectively.

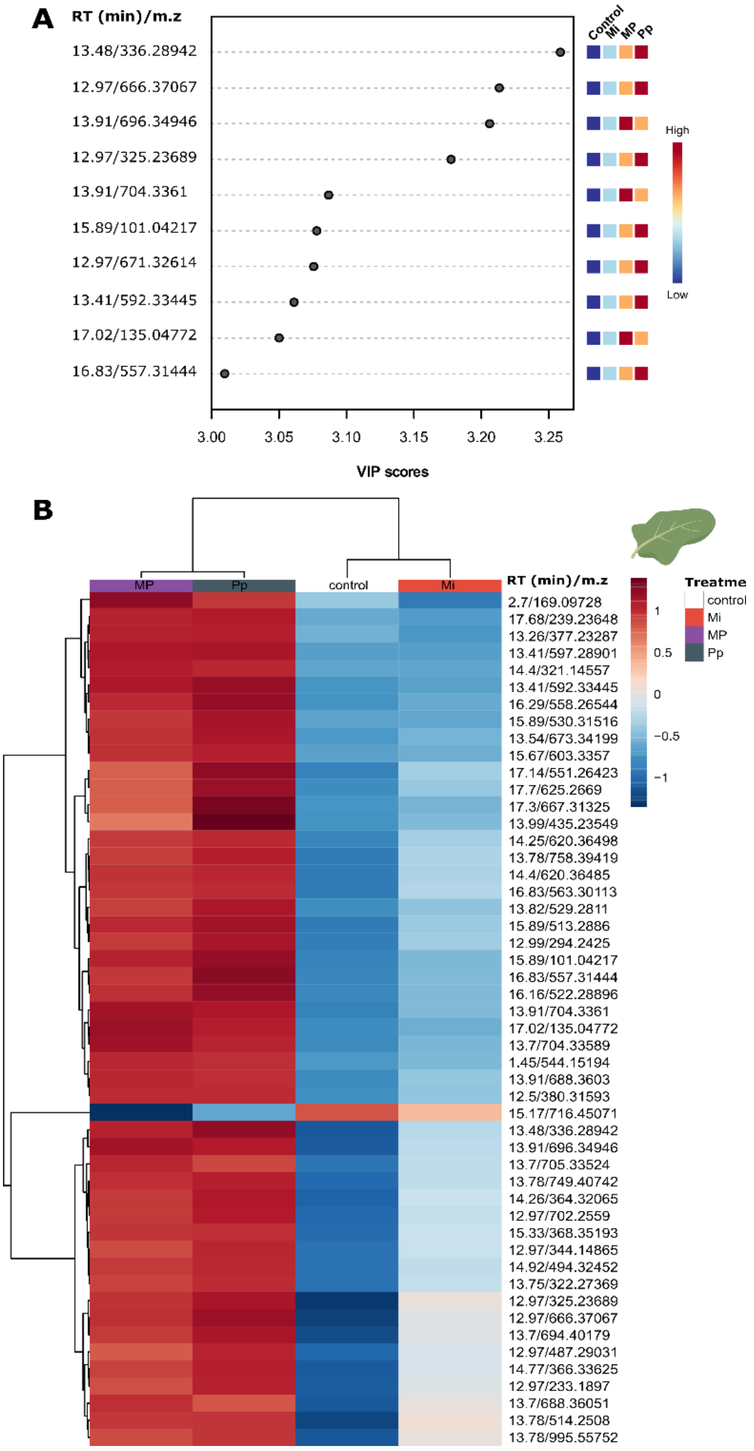

Fig. S2 (A) Top10 and (B) top 50 variable importance in projection (VIP) on the first component after Partial Least squares-discriminant analysis (PLS-DA) of the untargeted leaf metabolome data, 10dpni. Heatmap depicts feature abundance based on peak intensities. Clustering was based on Euclidean distance and Ward algorithm methods. Mi= *M. incognita*-infected, Pp= *P. penetrans*-infected, MP= concurrent Mi and Pp-infected plants.

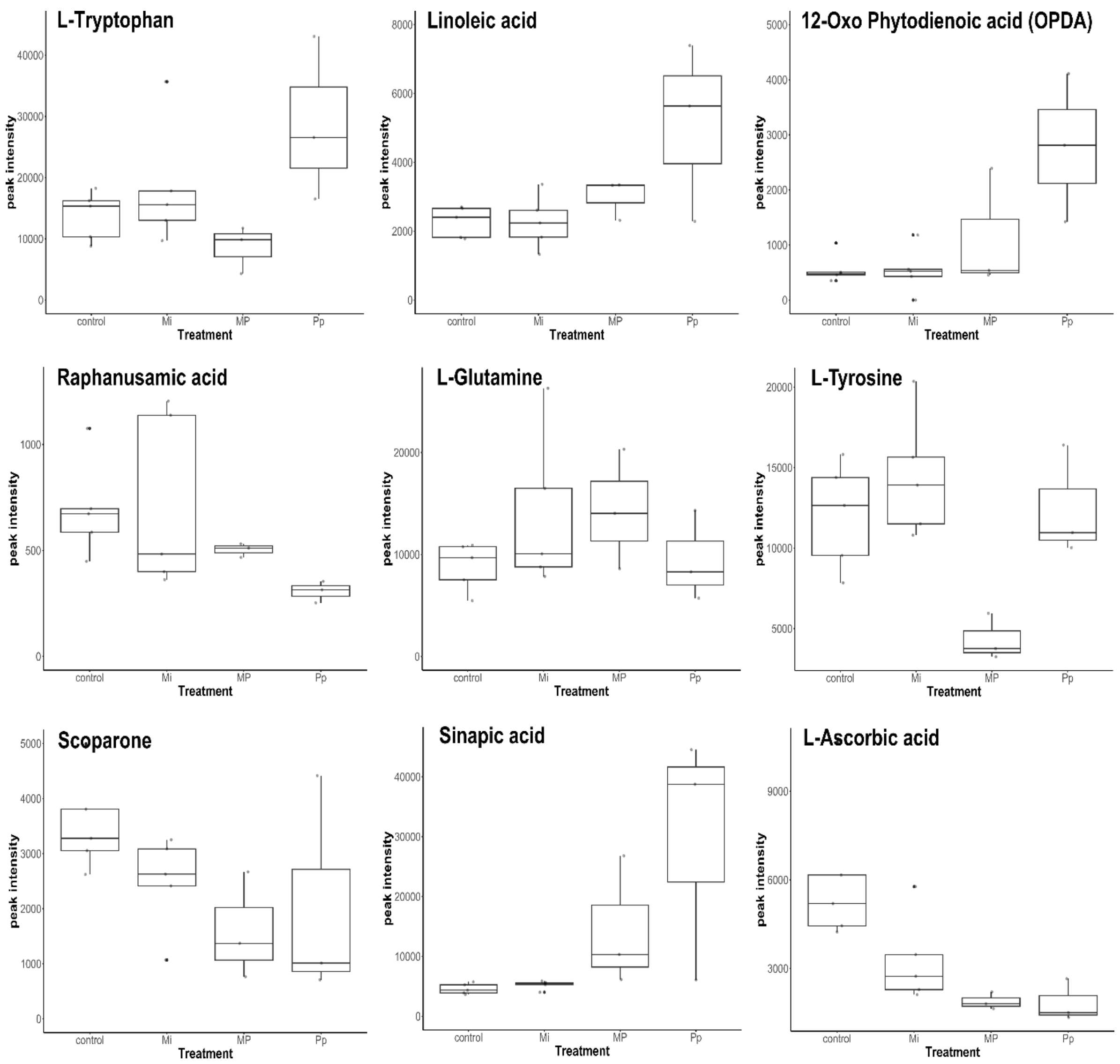

Fig. S3. Boxplots of differentially abundant (DA) features in the leaves of nematode-infected (Mi, MP, Pp) plants versus uninfected control plants. Points represent the number of biological replicates per treatment ($n_{\text{Mi and control}} = 5$; $n_{\text{MP and Pp}} = 3$). Abbreviations: Mi= *Meloidogyne incognita*, Pp= *Pratylenchus penetrans*, MP= *M. incognita* + *P. penetrans*.

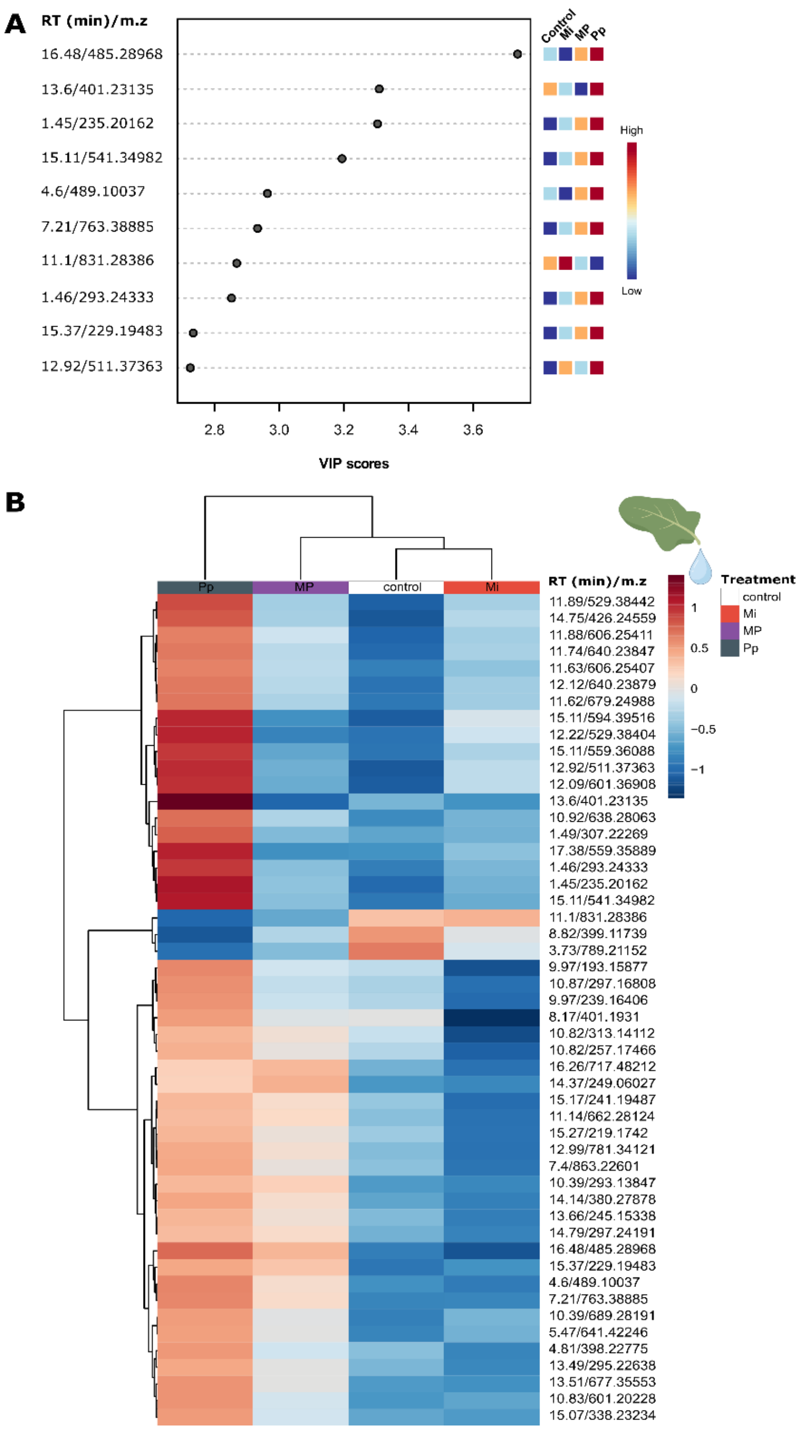

Fig. S4. (A) Top10 and (B) top 50 variable importance in projection (VIP) on the first component after Partial Least squares-discriminant analysis (PLS-DA) of the untargeted phloem metabolome data at 10dpni. Heatmap depicts feature abundance based on peak intensities. Clustering was based on Euclidean distance and Ward algorithm methods. Mi= *M. incognita*-infected, Pp= *P. penetrans*-infected, MP= concurrent Mi and Pp-infected plants.

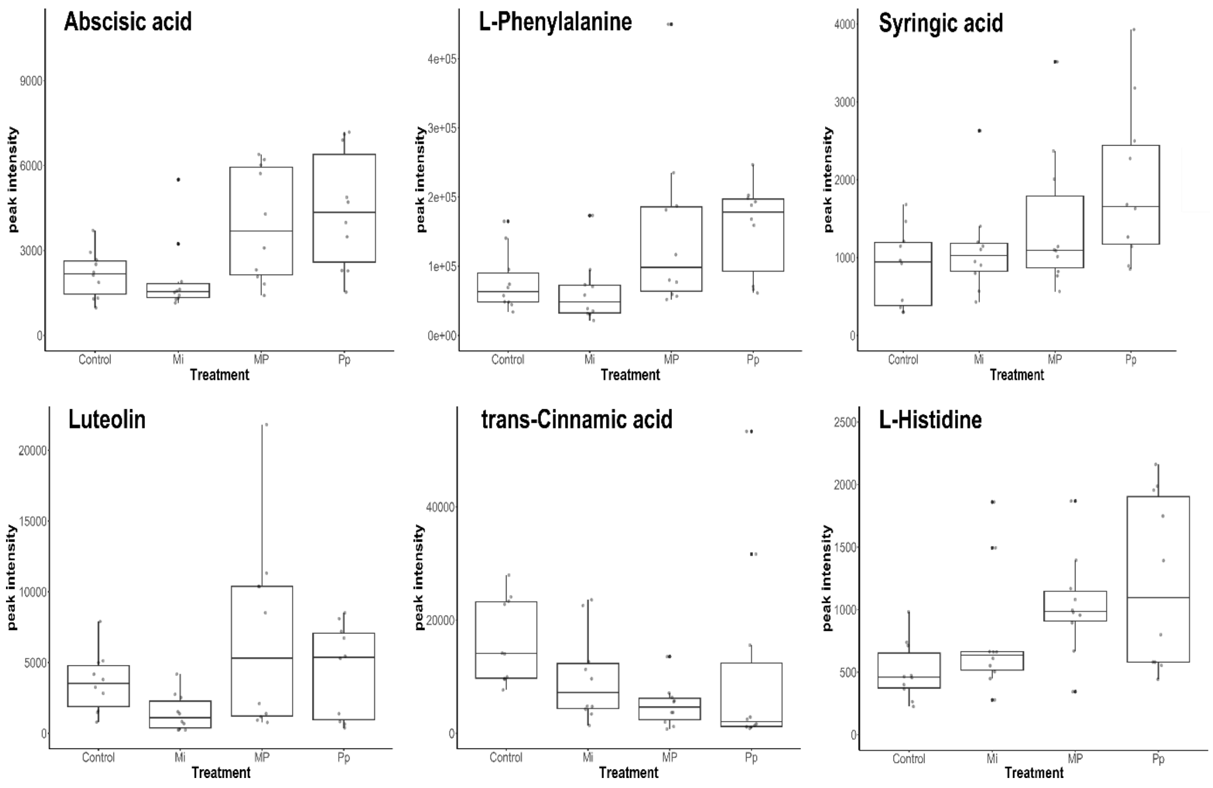

Fig. S5. Boxplots of differentially abundant (DA) features in the phloem of nematode-infected (Mi, MP, Pp) plants versus uninfected control plants. Points represent the number of biological replicates per treatment (n =10). Abbreviations: Mi= *Meloidogyne incognita*, Pp= *Pratylenchus penetrans*, MP= *M. incognita* + *P. penetrans*.

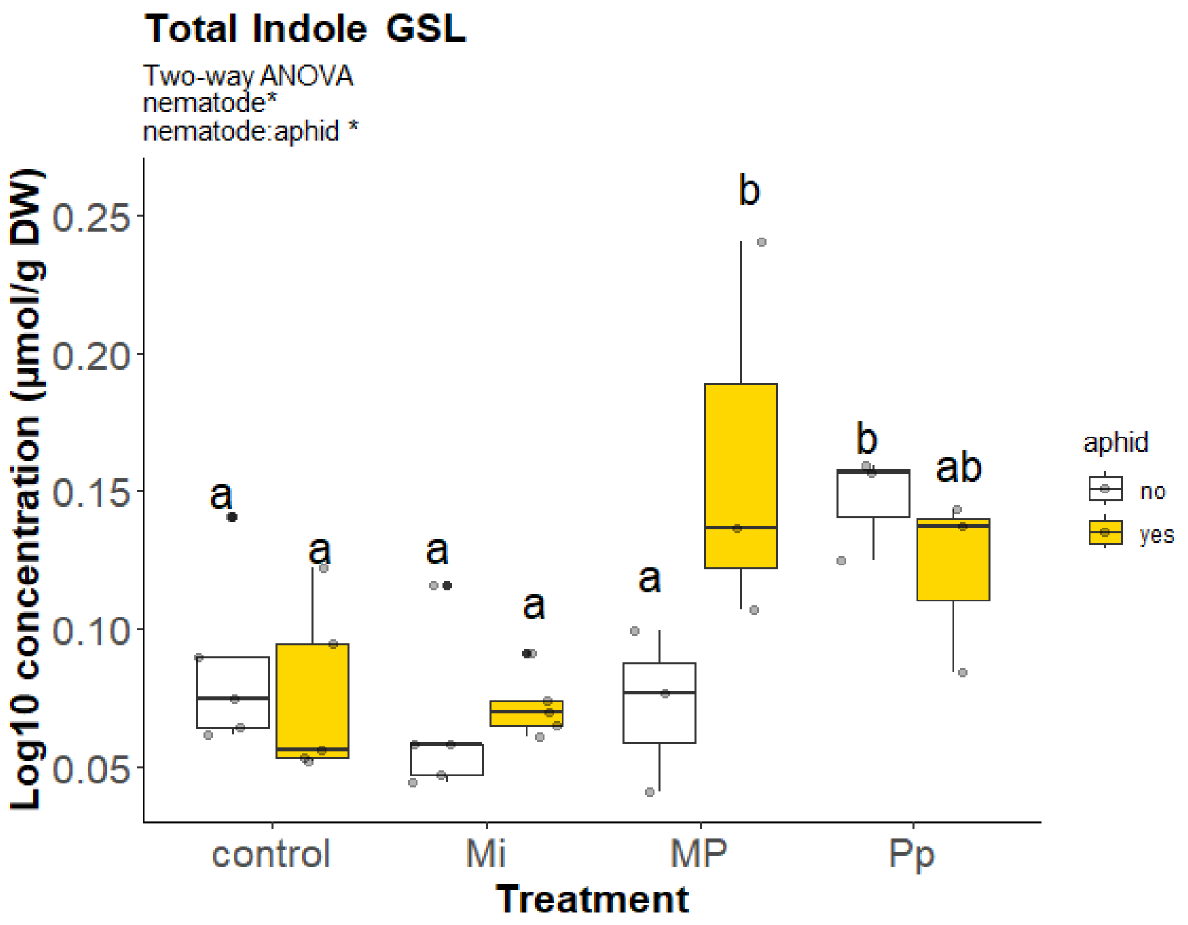

Fig. S6. Total indole glucosinolate (4-hydroxyglucobrassicin + glucobrassicin) concentrations in *Brassica nigra* leaves from plants infected with either individual, or combinations of nematodes (Mi, MP, Pp) and aphids (*Brevicoryne brassicae*). Points represent the number of biological replicates per group (n Mi and control = 5; n MP and Pp = 3). Boxplots labelled with different letters are significantly different as per pairwise t-test at $p<0.05$. Abbreviations: Mi= *Meloidogyne incognita*, Pp= *Pratylenchus penetrans*, MP= *M. incognita* + *P. penetrans*.

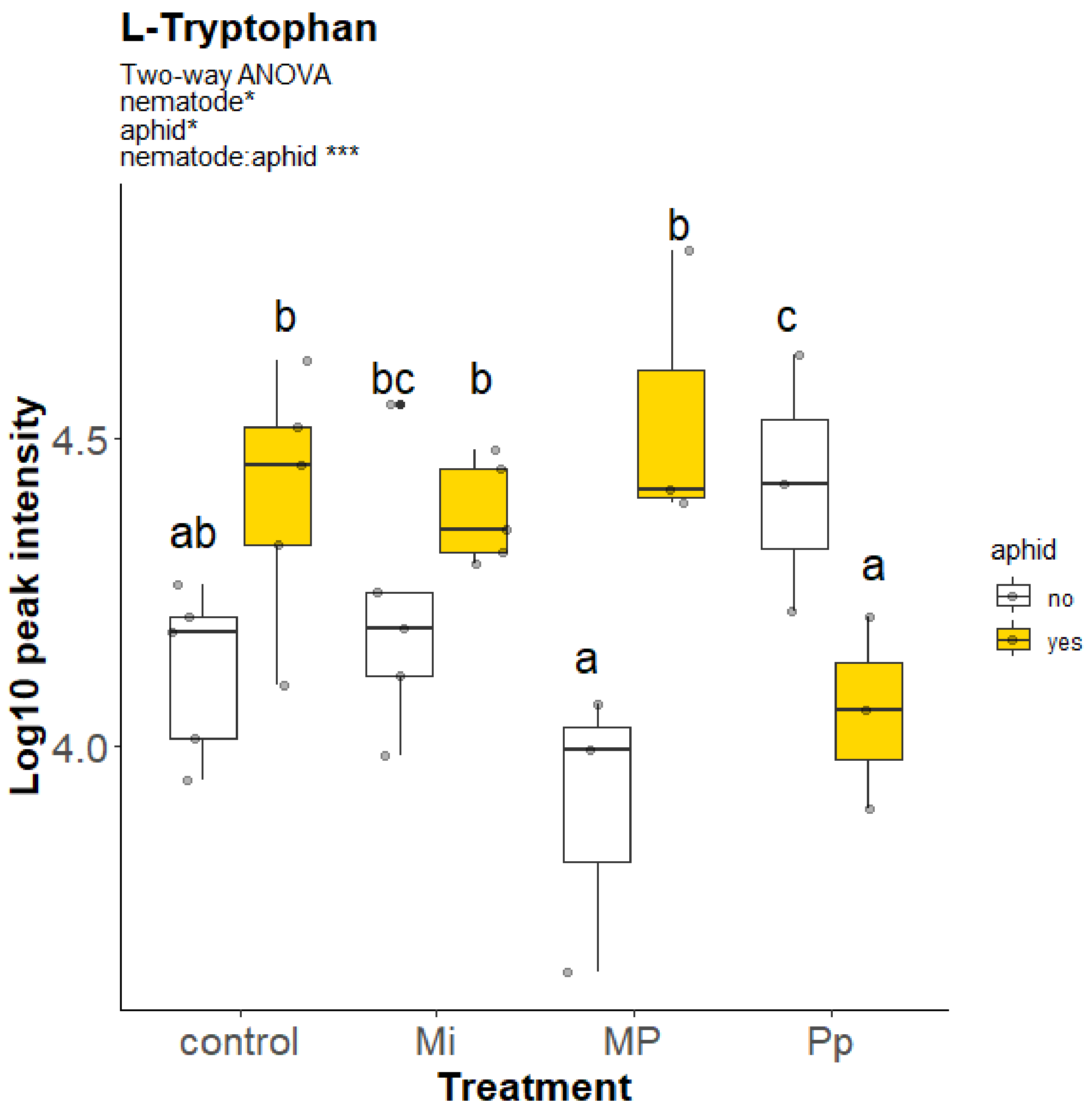

Fig. S7. L-tryptophan peak intensities as captured by untargeted metabolomics of *Brassica nigra* leaves from plants infected with either individual, or combinations of nematodes (Mi, MP, Pp) and aphids (*Brevicoryne brassicae*). Points represent the number of biological replicates per group (n Mi and Control = 5; n MP and Pp = 3). Boxplots labelled with different letters are significantly different as per pairwise t-test at $p<0.05$. Abbreviations: Mi= *Meloidogyne incognita*, Pp= *Pratylenchus penetrans*, MP= *M. incognita* + *P. penetrans*.

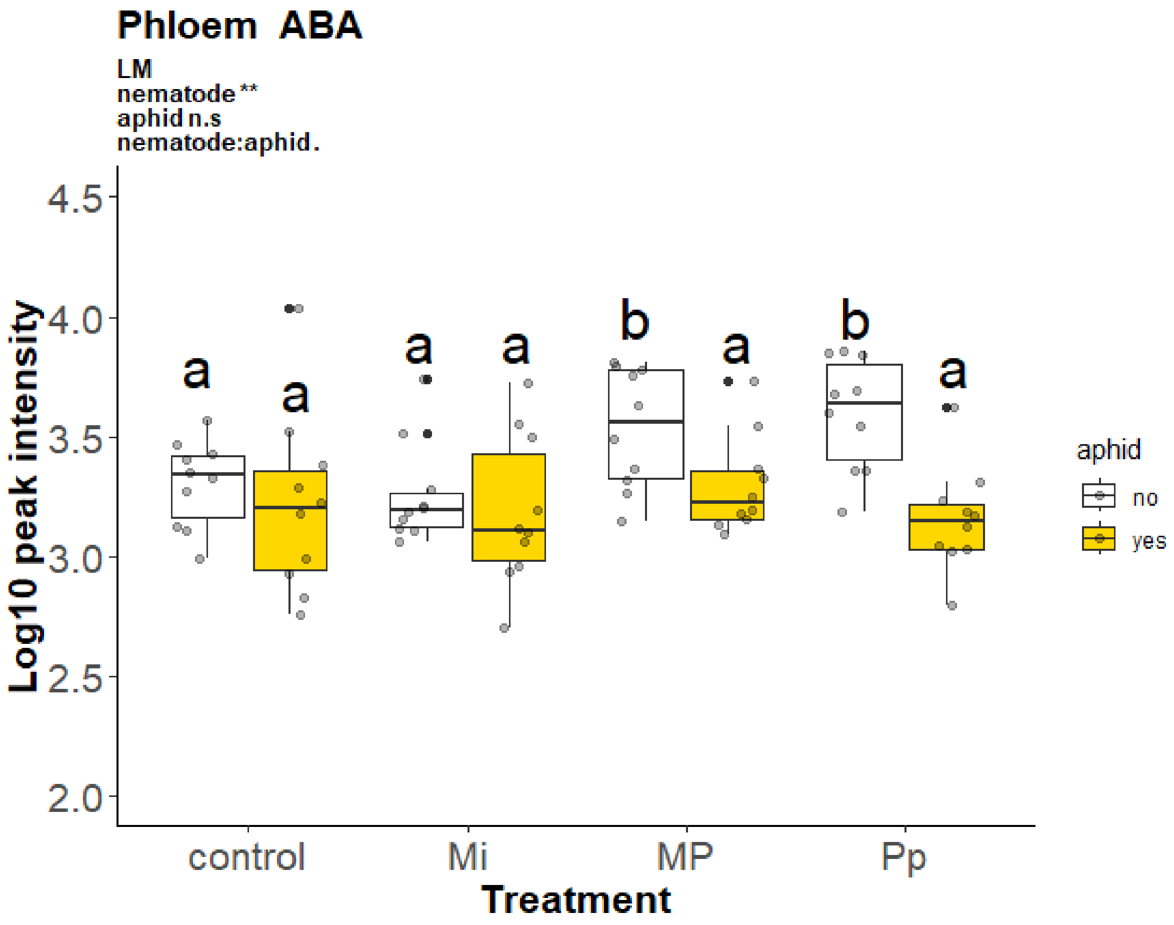

Fig. S8. Abscisic acid (ABA) peak intensities as captured by untargeted metabolomics of *Brassica nigra* phloem from plants infected with either individual, or combinations of nematodes (Mi, MP, Pp) and aphids (*Brevicoryne brassicae*). Points represent the number of biological replicates per group (n =10). Boxplots labelled with different letters are significantly different as per pairwise t-test at p<0.05. Abbreviations: Mi= *Meloidogyne incognita*, Pp= *Pratylenchus penetrans*, MP= *M. incognita* + *P. penetrans*.

Table S1. Classified leaf and phloem feature tables and list of differentially abundant leaf and phloem metabolites (separate excel file)

Table S2. Results of MANOVA and univariate analyses of phytohormones from the leaves of Brassica nigra plants infected with nematodes and aphids

**MANOVA** Phytohormones						
	**Df**	**Pillai's Trace**	**approx F**	**Num Df**	**Den Df**	**Pr(>F)**
Intercept	1	0.990	488.266	4	19	**2.22e-16**
Nematodes	3	1.138	3.212	12	63	**0.0012**
Aphids	1	0.131	0.718	4	19	0.5901
Nematodes:Aphids	3	0.927	2.347	12	63	**0.0146**
**Univariate ANOVA**						
			**F**	**DFn**	**DFd**	**Pr(>F)**
**ABA**						
	Nematodes		2.093	3	22	**0.0012**
	Aphids		5.802	1	22	0.5901
	Nematodes:Aphids		3.966	3	22	**0.0146**
**SA**						
	Nematodes		4.943	3	22	**0.0089**
	Aphids		6.791	1	22	**0.0161**
	Nematodes:Aphids		2.332	3	22	0.1019
**JA**						
	Nematodes		1.504	3	22	0.2413
	Aphids		0.324	1	22	0.5748
	Nematodes:Aphids		0.622	3	22	0.6084
**Ja-Ile**						
	Nematodes		0.276	3	22	0.8423
	Aphids		3.709	1	22	0.0671
	Nematodes:Aphids		1.084	3	22	0.3764

Table S3. Results of MANOVA and univariate analyses of glucosinolates from the leaves of Brassica nigra plants infected with nematodes and aphids

**MANOVA Glucosinolates**						
	**Df**	**Pillai's Trace**	**approx F**	**Num Df**	**Den Df**	**Pr(>F)**
Intercept	1	0.756	22.796	3	22	**6.05e-07**
Nematodes	3	1.138	0.766	9	72	**0.0082**
Aphids	1	0.073	0.575	3	22	0.6377
Nematodes:Aphids	3	0.868	3.254	9	72	**0.0023**

**Univariate ANOVA**					
		**F**	**DFn**	**DFd**	**Pr(>F)**
**Sinigrin**					
	Nematodes	0.079	3	24	0.9707
	Aphids	0.161	1	24	0.6919
	Nematodes:Aphids	1.399	3	24	0.2672
**4-Hydroxyglucobrassicin**					
	Nematodes	15.358	3	24	**8.62e-06**
	Aphids	0.046	1	24	0.8312
	Nematodes:Aphids	13.724	3	24	**2.04e-05**
**Glucobrassicin**					
	Nematodes	2.539	3	24	0.2409
	Aphids	2.636	1	24	0.0989
	Nematodes:Aphids	1.592	3	24	0.1659

Table S4. Results of PERMANOVA and univariate analyses of compound superclass from the untargeted phloem metabolome data.

PERMANOVA results						
	Df	SumOfSqs	R^2	F	Pr(>F)	
**nema**	3	0.15696	0.05417	3.1927	**0.013**	
**aphid**	1	0.05729	0.01977	3.496	**0.038**	
**day**	1	0.07352	0.02537	4.4861	**0.021**	
nema:aphid	3	0.07529	0.02598	1.5314	0.165	
nema:day	3	0.04753	0.0164	0.9668	0.428	
aphid:day	1	0.01865	0.00644	1.1381	0.273	
nema:aphid:day	3	0.10844	0.03743	2.2058	0.056	
Residual	144	2.35981	0.81443			
Total	159	2.89749	1			
Univariate analysis, lm(formula = DV ~ nema * aphid * day)						
	Df	Multiple R^2	Adjusted R^2	F	Pr(>F)	
**Alkylresorcinols**	15	0.2314	0.1513	2.89	**0.0005302**	***
Amino acid glycosides	15	0.1354	0.04538	1.504	0.1109	
**Aminosugars and aminoglycosides**	15	0.2122	0.1301	2.586	**1.86E-03**	**
**Anthranilic acid alkaloids**	15	0.1869	0.1022	2.206	**0.008527**	**
**Apocarotenoids**	15	0.1569	0.06905	1.786	**0.0418**	*
**Aromatic polyketides**	15	0.2556	0.178	3.296	**0.00009682**	***
Carotenoids (C40)	15	0.1496	0.06098	1.688	5.93E-02	.
**Chromanes**	15	0.2109	0.1288	2.566	**0.002011**	**
**Coumarins**	15	0.2609	0.1839	3.389	**0.00006537**	***
**Cyclic polyketides**	15	0.2144	0.1326	2.62	**0.001613**	**
Diarylheptanoids	15	0.08634	-0.008829	0.9072	5.58E-01	
**Diterpenoids**	15	0.2229	0.1419	2.753	**0.0009349**	***
**Eicosanoids**	15	0.372	0.3066	5.686	**4.724E-09**	***
**Fatty Acids and Conjugates**	15	0.2008	0.1175	2.412	**0.003761**	**
**Fatty acyl glycosides**	15	0.2165	0.1349	2.653	**1.41E-03**	**
Fatty acyls	15	0.08366	-0.01179	0.8764	0.5916	
**Fatty amides**	15	0.1703	0.08383	1.97	**0.0212**	*
Fatty esters	15	0.1326	0.04221	1.467	0.125	
Flavonoids	15	0.1508	0.0623	1.704	5.60E-02	.
**Glycerolipids**	15	0.2129	0.131	2.597	**0.001773**	**
**Glycerophospholipids**	15	0.2109	0.1287	2.566	**0.002013**	**
**Guanidine alkaloids**	15	0.316	0.2447	4.435	**8.038E-07**	***
**Histidine alkaloids**	15	0.2056	0.1228	2.484	**2.81E-03**	**
**Isoflavonoids**	15	0.1773	0.09165	2.07	**0.0145**	*
Lignans	15	0.1491	0.0605	1.683	0.06048	.
**Linear polyketides**	15	0.3832	0.319	5.965	**1.549E-09**	***
**Lysine alkaloids**	15	0.2476	0.1693	3.16	**1.72E-04**	***
Meroterpenoids	15	0.1127	0.02022	1.219	0.2641	
**Monoterpenoids**	15	0.2783	0.2032	3.702	**0.00001744**	***
**Naphthalenes**	15	0.2198	0.1385	2.704	**0.001142**	**
Nicotinic acid alkaloids	15	0.09828	0.004354	1.046	0.412	
**Nucleosides**	15	0.2628	0.186	3.421	**5.70E-05**	***
**Octadecanoids**	15	0.2374	0.1579	2.988	**0.0003521**	***
**Oligopeptides**	15	0.1949	0.111	2.324	**0.005356**	**
Ornithine alkaloids	15	0.1345	0.04439	1.492	0.1152	
**Peptide alkaloids**	15	0.2268	0.1463	2.816	**0.00072**	***
Phenanthrenoids	15	0.1434	0.05418	1.607	0.07848	.
**Phenolic acids (C6-C1)**	15	0.2171	0.1356	2.662	**0.001357**	**
**Phenylethanoids (C6-C2)**	15	0.2408	0.1617	3.045	**0.0002775**	***
**Phenylpropanoids (C6-C3)**	15	0.196	0.1122	2.34	**0.005022**	**
**Phloroglucinols**	15	0.2364	0.1569	2.972	**0.0003756**	***
**Polycyclic aromatic polyketides**	15	0.2779	0.2027	3.694	**1.81E-05**	***
**Polyethers**	15	0.3503	0.2827	5.177	**3.73E-08**	***
**Polyols**	15	0.1662	0.07935	1.914	**0.02618**	*
**Pseudoalkaloids**	15	0.1748	0.08879	2.033	**0.01669**	*
**Pseudoalkaloids (transamidation)**	15	0.223	0.1421	2.756	**0.0009236**	***
**Saccharides**	15	0.5858	0.5427	13.58	**< 2.2e-16**	***
**Sesquiterpenoids**	15	0.2353	0.1557	2.954	**0.0004049**	***
Small peptides	15	0.1489	0.06027	1.68	0.06105	.
**Sphingolipids**	15	0.3275	0.2575	4.676	**2.95E-07**	***
**Steroids**	15	0.1699	0.08347	1.965	**0.02157**	*
**Stilbenoids**	15	0.294	0.2205	3.998	**5.02E-06**	***
Tetramate alkaloids	15	0.1355	0.04547	1.505	0.1106	
**Triterpenoids**	15	0.3951	0.3321	6.27	**4.63E-10**	***
**Tryptophan alkaloids**	15	0.1868	0.1021	2.205	**0.008556**	**
**Tyrosine alkaloids**	15	0.1929	0.1089	2.295	**0.006001**	**
**Xanthones**	15	0.1669	0.08016	1.924	**0.02522**	*
**unclassified**	15	0.1641	0.07699	1.884	**0.02921**	*
